# Therapy Companion Mobile App for Acceptance and Commitment Therapy Exercises (ACTaide): Therapist and Client Co-Design Study

**DOI:** 10.2196/69532

**Published:** 2025-07-24

**Authors:** Serena Thapar, Daniela Quesada, Bärbel Knäuper

**Affiliations:** 1 Department of Psychology McGill University Montreal, QC Canada

**Keywords:** mental health, mobile app, focus group, co-design, user-centered design, acceptance and commitment therapy, psychotherapy

## Abstract

**Background:**

Acceptance and commitment therapy (ACT) relies heavily on the between-session practice of therapeutic exercises to promote skill acquisition and improve psychological flexibility. However, adherence to this between-session practice remains a challenge. Mobile apps offer a promising solution to bridge this gap. However, few ACT apps focus exclusively on supporting clients in their between-session practice, and fewer apps involve stakeholders in their design. ACTaide, a therapy companion mobile app co-designed with stakeholders, addresses these barriers by guiding clients through ACT exercises and metaphors using annotated image sequences, supporting their between-session practice.

**Objective:**

This study aimed to co-design ACTaide with therapists and clients, incorporating their feedback to ensure the app aligns with clinical goals and the needs of end users. The research explored stakeholder preferences and feedback on app functionality, design, and features to guide iterative design improvements.

**Methods:**

Using a qualitative, user-centered design framework, we conducted 4 consecutive focus groups: 2 with 10 licensed ACT therapists and 2 with 14 psychotherapy clients. Each focus group included semistructured discussions and co-design activities. Data were collected through audio recordings and design artifacts (eg, sketches), which were analyzed using thematic content analysis.

**Results:**

A total of 9 themes were identified, reflecting areas of convergence and divergence between therapists and clients. The therapists and clients expressed enthusiasm for ACTaide as a tool to support between-session practice. Both groups emphasized the importance of a user-friendly, intuitive, and aesthetically appealing interface, with a preference for high-quality visuals over text-heavy features. Personalization and customization were viewed as essential for enhancing app engagement. The therapists prioritized accessibility and clinical appropriateness, voicing concerns about features that may be inconsistent with ACT principles, such as symptom rating scales, and clarified their role in app delivery. By contrast, the clients emphasized wanting greater interactivity and elements of gamification to improve engagement. Slight discrepancies were noted between therapists’ preferences for minimal designs and clients’ preferences for more vibrant and engaging aesthetics. Overall, both groups recognized the app’s potential to address barriers to homework adherence and to extend the benefits of therapy into clients’ daily lives.

**Conclusions:**

The study illustrates the value of using a user-centered, co-design approach in the development of ACTaide, an adjunctive mental health app for the between-session practice of ACT exercises and metaphors tailored to therapist and client preferences. Through the integration of stakeholder feedback, the findings provide actionable insights for designing psychotherapy tools that balance clinical goals with user preferences. Future research will focus on testing high-fidelity prototypes to evaluate acceptability, usability, and engagement.

## Introduction

### Background

Mental health problems are a global concern, representing a leading cause of disability [1]. Among evidence-based psychotherapies, acceptance and commitment therapy (ACT) has been found to be efficacious across a wide range of conditions, including anxiety and depression [2], the most common mental health problems worldwide [1]. For those who have access to psychotherapy, it has been reported that clients do not fully engage in homework, with surveys indicating that approximately 20% to 50% of clients do not engage in any between-session practice [3]. Homework (ie, between-session practice of skills taught in therapy) has long been considered central to the effectiveness of psychotherapy across a wide variety of clinical conditions [3-5], with meta-analyses showing large effect sizes of homework quantity and quality on therapy effectiveness [6,7]. Through engaging in skill acquisition exercises, homework extends the therapeutic benefits of treatment beyond therapy sessions, thereby promoting enhanced symptom improvement [8,9]. Unfortunately, there is a lack of therapeutic tools that focus specifically on encouraging clients to practice exercises between sessions [10], including for ACT [11]. This study addresses this issue by developing a low-fidelity prototype of ACTaide, a therapy companion mobile app aimed at helping clients practice ACT exercises between sessions via visually illustrated and annotated ACT exercise and metaphor sequences.

### ACT Model

As part of the third wave cognitive behavioral therapies, ACT combines mindfulness, acceptance, and behavior change processes to enhance psychological flexibility [12]. At the core of healthy emotional functioning, psychological flexibility refers to the ability to live in the present moment and be open to experiences for the purpose of engaging in values-guided actions [13]. It uses psychoeducation, experiential exercises, and metaphor exercises to positively impact clinical outcomes.

The efficacy of ACT has been shown across a wealth of evidence. ACT has been extensively studied in >1000 randomized trials [14,15] that have been summarized in several meta-analyses [16,17] and a review of 20 meta-analyses [2], demonstrating medium-to-large effect sizes across a broad range of mental health conditions. The World Health Organization has adapted ACT in >25 languages to be used with and by people experiencing stressful circumstances [18]. ACT relies heavily on between-session practice of experiential and metaphorical exercises for psychological improvement [15,19]. However, ACT has not been exempted from the issue of insufficient homework engagement [11].

Psychological flexibility is the overarching target of ACT and is meant to be cultivated by developing the 6 skills or therapeutic processes specified in the ACT hexaflex theoretical model [4,20,21]. These 6 processes are *acceptance* (ie, learning to accept difficult thoughts and feelings rather than trying to fight or avoid them), *cognitive defusion* (ie, learning to gain a psychological distance from thoughts and feelings), *present-moment awareness* (ie, learning to maintain awareness of current thoughts, emotions, sounds, and sights), *self-as-context* (ie, learning to observe internal experiences from a distance), *values* (ie, learning to identify and live by deeply held personal values), and *committed action* (ie, learning to act or not act in line with these values)*.*

To foster the development of psychological flexibility, ACT uses metaphors and experiential exercises that target the 6 processes [12,13,22]. Metaphors have been long-standing therapeutic devices in clinical settings and across therapeutic interventions [23,24]. Their easy, story-like properties enable clients’ understanding of their personal circumstances while directing them to solutions that could promote positive behavior change [23,25]. Finally, metaphors allow clients to get some distance from their own difficult thoughts, thus preventing the occurrence of cognitive fusion (ie, the opposite of defusion) and enhancing psychological flexibility [23]. ACT’s emphasis on experiential learning is also demonstrated through its use of experiential exercises. These exercises assist clients in facing psychological processes, such as thoughts, feelings, memories, and sensations that they have previously avoided, by encouraging experiential engagement with them [26]. Experiential exercises also serve as a vehicle for skill acquisition [4]. What remains a challenge is clients’ engagement with such exercises as part of homework.

Homework has long been considered central to the effectiveness of psychotherapy, with meta-analyses showing large effect sizes of homework quantity and quality on therapy effectiveness [5,6,27]. However, it is not uncommon for psychotherapy patients to engage minimally in between-session practice of exercises learned during therapy sessions [3,28]. Reasons for low engagement include low motivation, not remembering the details of the exercises, not fully understanding them, or not having access to the exercises when needed [29-31].

### Existing ACT Mobile Apps

Mental health apps have prospered in the past decade, with >15,000 on the market [32]. They have gained attention for their potential to enhance skills training in ACT [33,34]. More than 20 ACT apps exist, displaying considerable heterogeneity in their purpose and features [35-56]. Many of these apps rely primarily on lengthy psychoeducational content delivered via text and video, which generally has been found to hinder user engagement within mental health apps [57]. Furthermore, to the best of our knowledge, no existing app focuses exclusively on the between-session practice of ACT metaphors and exercises.

Low engagement is a pervasive issue within mental health apps [58], including those focused on ACT [11,49,59,60]. While reasons for this poor engagement remain unclear, researchers posit that app design often fails to adequately consider users’ experiences and needs [58,61,62]. Among the existing ACT apps, few have incorporated participatory approaches during development [41-43,52,54], such as co-design [52]. While many ACT apps show promise in research settings [43,52,54], their access is limited, given that many of them were created for research endeavors and are not publicly available [37-39,41-44,47-49,52-54]. This limitation is particularly significant given the interest expressed by practitioners in integrating ACT apps into practice. For example, results of a survey of 356 members of the Association for Contextual Behavioral Science revealed that ACT practitioners expressed strong interest in ACT apps and would find them beneficial for practicing ACT skills between sessions [63]. Indeed, participants in an ACT workshop reported wanting a mobile app to access learned skills as a support for implementation in their daily lives [64].

### ACTaide

This study will address this issue by developing and evaluating a smartphone app, ACTaide, that clients can use to practice ACT exercises between sessions. The app will include the most commonly used ACT exercises and metaphors, such as those identified in *The Big Book of ACT Metaphors* [13] and *ACT Made Simple* [65]. Unlike existing apps, ACTaide offers a novel delivery of ACT exercises and metaphors to be used exclusively for between-session practice of homework. The proposed smartphone app will visually illustrate the processes as annotated image sequences that guide patients step-by-step through exercises and metaphors that reflect the therapeutic processes of ACT. Each image will be accompanied by a brief text-based annotation.

The use of images and visuals has been documented to improve retention and comprehension [66]. For example, a meta-analysis of the effects of graphics on reading comprehension found that the combined use of both image and text was particularly beneficial for understanding text compared to the use of words alone [67]. Empirical evidence from medical education shows that images are more easily processed than words, are easier to recall, and have better retention than text alone [68-72]. For example, annotated image sequences on how to take medication have been shown to lead to enhanced recall, comprehension, and medication adherence [70,73].

### User-Centered Design

Clients’ current low engagement with mental health apps has prompted researchers to move away from developing mental health apps in isolation from end users. Instead, the user-centered design framework, an evidence-based approach that emphasizes the involvement of end users in the design process of mobile apps, has gained attention [74]. This approach has been demonstrated to be effective for the development of adjunctive mobile apps, in addition to being recommended by the World Health Organization to maximize outcomes such as engagement [75,76]. Therefore, the development of ACTaide is guided by the user-centered design framework that is built upon 2 frameworks: the information system framework (ie, user-centered design process) and the design thinking framework (ie, user-centered solution-focused approach) [75]. The information system framework emphasizes an iterative approach where understanding end users’ needs, researching, learning from feedback, and integrating their insight into the design are central to the app development process. Design thinking, on the other hand, underscores the importance of empathy and creativity to further understand end users’ needs. Both frameworks enable the creation of the app through a collaborative development process informed by relevant stakeholders, a process that has been successfully used for mental health app development [77-79].

### Aims and Research Question

**Figure 1:**
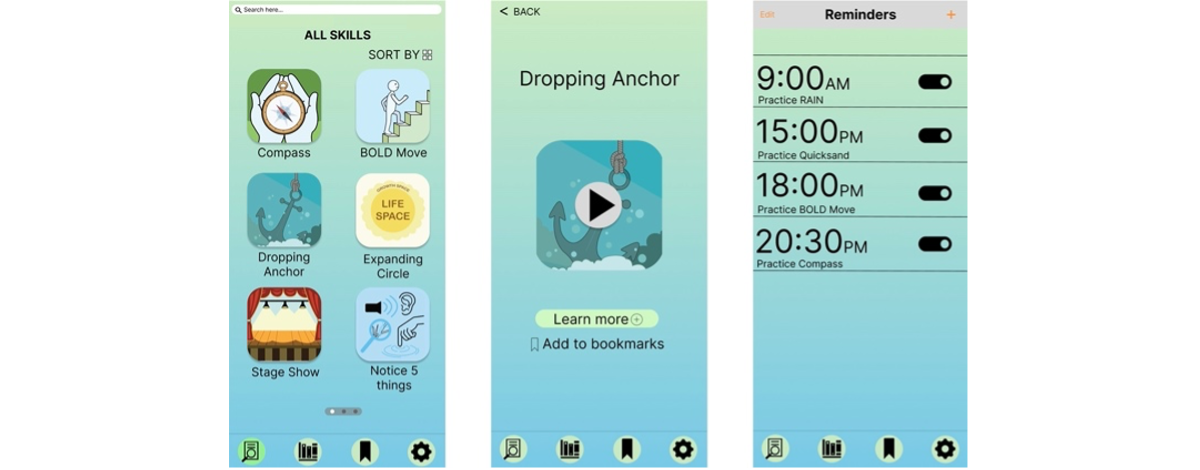
Example low-fidelity wireframes of ACTaide, depicting the basic layout and content of the home page, start exercise page, and reminders page. These were presented to the final focus group of the clients after incorporating feedback from previous focus groups as part of an iterative design process.

This qualitative research study will inform the first aim of this research project, which is to incorporate the feedback of end users into the smartphone app design process. The study aims to answer the following research question: “What is therapists’ and clients’ feedback on a low-fidelity wireframe prototype of ACTaide, a therapy companion smartphone app for visually delivering ACT exercises and metaphors?” Specifically, *feedback* refers to opinions, preferences, and perceptions regarding the prototype’s interface, features, content, and overall usability. In this context, a *low-fidelity wireframe* refers to an initial, basic, and noninteractive visual representation of the ACTaide smartphone app (Figure 1). This wireframe is a simplified, static draft of the app’s layout that outlines its visual structure and content, serving as a starting point for design and development without full functionality or graphics.

## Methods

### Study Design

To engage relevant stakeholders in a user-centered design process, data for this research were collected through focus groups, a widely recognized method that encourages discourse among participants on clearly defined topics [80]. Focus groups were selected for this study because of their effectiveness in eliciting diverse opinions and perceptions from therapists and patients involved in the co-design of the ACTaide smartphone app. This methodology has been successfully applied in previous mental health app development research [78]. Focus groups allow the exploration of both converging and divergent views, offering rich insights into participants’ feedback and enhancing our understanding of their experiences. A total of 4 focus groups were conducted between January 2024 and October 2024: the first 2 with therapists and the subsequent 2 with clients. The focus groups were sequential, meaning that feedback from each session informed adjustments to the ACTaide wireframes before subsequent focus groups (Multimedia Appendix 1), allowing an iterative development process. The sequential order ensured that client input directly built upon a framework already vetted by clinical professionals, resulting in a well-integrated and clinically accurate tool. A summary of design changes made based on feedback during each focus group can be found in Multimedia Appendix 2.

### Participants

Recruitment for therapist participants was conducted using purposive sampling to align with the research project’s needs for clinical input [81]. To create a pool of potential participants to recruit from, we randomly selected and contacted 200 psychotherapists listed on *Psychology Today* (100 in Quebec and 100 in Ontario) [82] who identified as offering mindfulness-based therapy or ACT. Of these, 33.5% (67/200) expressed interest in contributing to the app development process. Therapists who worked in Montreal or the Greater Montreal Area were prioritized for recruitment to allow in-person attendance. Additional therapists in Montreal were identified through web searches and snowball sampling. For this pool, eligibility criteria included a minimum of 5 years of ACT experience, being proficient in English, and being a licensed practitioner. Therapists were invited to participate in the study via email. Using purposive sampling, 10 therapists were selected to represent a diversity of age, race, and gender (refer to Table 1 for demographic details). A total of 2 focus groups (5 in each group) were conducted.

Client participants, who represented the other stakeholder group, were recruited through flyers distributed on social media and web forums. All participants were adults (aged ≥18 years) with previous psychotherapy experience that included assigned between-session exercises. All participants were proficient in English and able to attend an in-person focus group in Montreal. The participants completed a screening questionnaire to determine eligibility and were then contacted via email with an invitation to participate in the study. Quota sampling was used to ensure representation across age ranges and gender, consistent with psychotherapy client demographics [83]. A total of 2 focus groups (8 attendees in the first group and 6 in the second group) were conducted (refer to Table 2 for demographic details).

**Table 1:** Demographic data of the therapists (N=10).

Demographic variables and categories		Participants, n (%)
**Gender**		
	Men	3 (30)
	Women	7 (70)
**Age (y)**		
	29-45	4 (40)
	≥46	6 (60)
**Years of experience with ACT^a^**		
	5-10	7 (70)
	≥10	3 (30)
**Years of clinical experience**		
	<15	5 (50)
	≥15	5 (50)
**Primary practice location**		
	Hospital (outpatient)	4 (40)
	Private practice	4 (40)
	Other	2 (20)
**Clinical position**		
	Doctoral-level psychologist	8 (80)
	Master’s-level social worker	2 (20)
**Ethnicity**		
	European	8 (80)
	Hispanic or Latinx	2 (10)
	Southeast Asian	1 (10)

^a^ACT: acceptance and commitment therapy.

**Table 2:** Demographic data of the psychotherapy clients (N=14).

Demographic variables and categories		Participants, n (%)
**Gender**		
	Men	6 (43)
	Women	8 (57)
**Age (y)**		
	18-29	5 (36)
	30-39	2 (14)
	40-49	4 (29)
	50-59	2 (14)
	≥60	1 (7)
**Education**		
	Some college, CEGEP^a^ (Quebec), or university	6 (43)
	Bachelor’s degree	5 (36)
	Graduate degree (master’s or PhD)	2 (14)
	High school diploma	1 (7)
**Employment status**		
	Unemployed	3 (21)
	Employed or self-employed	5 (36)
	Undergraduate student	3 (21)
	Graduate student	3 (21)
**Ethnicity**		
	African	1 (7)
	European	9 (64)
	Hispanic or Latinx	1 (7)
	Middle Eastern	1 (7)
	South Asian	2 (14)
**Exposure to ACT^b^**		
	Yes	4 (29)
	Unsure	6 (43)
	No	4 (29)
**Therapy format**		
	Individual (one-on-one)	10 (71)
	Individual and group	4 (29)

^a^CEGEP: Collège d'enseignement général et professionnel (General and Professional College).

^b^ACT: acceptance and commitment therapy.

### Procedure

#### Overview

We first conducted 2 focus groups with 10 therapists, followed by 2 with 14 clients, ensuring sufficient group size to facilitate interactive discussions without being too large and disorganized. This focus group size aligns with the recommendations of 5 to 8 participants per group [84] and reflects the average sizes found in similar research [85]. Focus group sessions were held at the McGill Health Psychology Lab, with the exception of the second therapist group, which took place via videoconferencing. Each session consisted of three parts: (1) a structured background presentation, (2) a focus group discussion, and (3) a co-design session for clients. Each session lasted approximately 120 minutes.

#### Phase 1: Exploring Users’ Perceptions of ACTaide

The moderator presented an overview of ACTaide’s rationale and purpose. Participants shared feedback on the concept of a therapy companion mobile app for ACT exercises and metaphors to be practiced between sessions.

#### Phase 2: Understanding Participants’ Perspectives, Opinions, Preferences, and Concerns Related to ACTaide Wireframes

Participants reviewed all app wireframes, receiving a high-level description of each wireframe. While low-fidelity wireframes typically do not contain graphics, preliminary sketches of 3 annotated image sequences were presented to provide context for the exercise delivery format. Therefore, feedback on the preliminary sketches themselves was not solicited. Feedback on the design, features, and functionalities of wireframes in their entirety was solicited. After reviewing all wireframes, key questions prompted deeper insights into participants’ initial reactions, likes, dislikes, and suggestions for modifications.

#### Phase 3: Engaging Participants in a Co-Design Workshop

In the final segment, participants participated in a co-design session in which they sketched their ideal versions of the app.

The focus groups used semistructured interviewing techniques facilitated by a moderator (ST), who used predetermined opening, introductory, transition, key, and closing questions to stimulate conversation (Multimedia Appendix 3). The moderator-guided discussions related to wireframes while also adapting to emerging ideas. In total, 2 undergraduate researchers documented nonverbal behavior and group dynamics to support data analysis and capture emergent phenomena. All sessions were audio recorded and transcribed for analysis, with photographs taken of physical artifacts (eg, sketches from the co-design session).

### Data Analysis

Data for this study were collected through audio recordings from each focus group session, which resulted in 4 transcripts. These transcripts were meticulously reviewed by the research team and cross-referenced with the audio recordings to ensure accuracy. To uphold methodological rigor, we used thematic analysis as the primary analytical approach [86]. This method is particularly suited for systematically identifying collective patterns within a large dataset through an inductive lens, while also considering the contextual nuances of participants’ contributions [87]. Thematic analysis enables researchers to remain open to the complexities that may arise during discussions, free from the constraints of a rigid conceptual coding framework. In addition, recorded observations of nonverbal behavior, group dynamics, and emerging themes were reviewed to further enrich the analysis. Physical artifacts, such as sketches created during client co-design sessions, were also coded and analyzed.

The six-phase framework for conducting thematic analysis guided our approach: (1) becoming familiar with the data, (2) generating initial codes, (3) searching for themes, (4) defining themes, (5) iteratively reviewing themes, and (6) writing up the results [86]. ST and DQ independently read and reread all transcripts, applying open coding to identify initial codes. Subsequent meetings facilitated the expansion and refinement of codes as new insights emerged, leading to the identification of themes through iterative reviews. To enhance the validity and reliability of our analysis, we discussed the emerging themes with a group of researchers from the McGill Health Psychology team who were familiar with this research area, incorporating their feedback into the final themes. Quotes were extracted from transcripts for therapists and clients to illustrate the main themes that emerged from the data.

We then used a case study methodology to compare feedback from distinct end-user groups—therapists and clients. This comparative analysis aimed to uncover converging and diverging opinions [88,89], revealing how the app could be optimally tailored to meet the diverse needs of all end users. This comparative perspective was intended to provide a nuanced understanding of the differing attitudes toward the low-fidelity wireframes among therapists and patients. Ultimately, this analysis will inform decisions regarding the app’s redesign based on shared and contrasting viewpoints.

### Ethical Considerations

This study received approval from the McGill Research Ethics Office (institutional review board; 22-11-068). All participants provided written consent before participating in the study. To ensure privacy and confidentiality, all transcripts were deidentified before analysis. Furthermore, demographic information was aggregated in the reporting of study data. All participants were compensated for their participation in the study. Those who participated in the therapist focus groups were compensated CAD $200 (US $146.15). Those who participated in the client focus groups were compensated CAD $80 (US $58.46).

## Results

### Overview

A total of 9 themes emerged from the focus group discussions. In total, 6 themes were common across both therapist and client focus groups: the value of ACTaide, aesthetic design preferences, ease of use, customization and personalization, app guidance, and app engagement and gamification. A total of 3 additional themes were unique to therapist focus groups: therapist involvement, accessibility, and clinical concerns.

### The Value of ACTaide

Both the therapists and the clients consistently acknowledged the inherent value of ACTaide as an adjunctive tool to facilitate between-session practice. The therapists particularly endorsed the app’s potential for supporting the continuity of psychotherapy beyond the therapy room, which they deemed necessary for meaningful change:

Therapy isn’t really about what happens in the office...[it’s] about what our clients are actually doing different outside of sessions...something that can support out-of-session practice really moves people forward toward their goals.

Therapist 1

Many therapists also expressed enthusiasm for the therapy companion mobile app and its use as an adjunct to their clinical practice:

I think the idea of a companion app versus a standalone thing is a kind of nice idea...We are not expecting the app to do the therapy.

Therapist 4

The clients similarly valued the concept of the app, with several expressing that they often struggle to engage in between-session practice of psychotherapy skills, citing the app’s potential to help them stay accountable and consistent. For example, 1 client reflected on their experience in therapy, stating the following:

I think it [referring to the app] is a really good idea...it’s easy to forget the exercises that you’re supposed to implement.

Client 3

In addition to the overall value of the app itself, both the clients and the therapists acknowledged the value of various app features. For example, both the therapists and the clients saw value in the app having a reminder feature, believing that it would promote greater app use:

I just forget these apps are on my phone...but if it sends me prompts during the day, maybe I would be more likely to do whatever it tells me to do.

Client 13

Similarly, both the therapists and the clients expressed their appreciation for a *favorites* feature that would provide the option to shortlist exercises for easy access.

A unique perspective that emerged from the therapists’ focus group was the recognition of the value of the app’s format (ie, using annotated image sequences) for delivering exercises and metaphors:

The fact that it’s image-based is really appealing...that’s the big thing that stood out.

Therapist 7

While the clients did not express contrasting opinions, they did not comment positively or negatively on the format of delivery.

### Aesthetic Design Preferences

Both the therapists and the clients offered critical feedback on the visual appeal of the app and offered suggestions for improvement. Most expressed the importance of an appealing aesthetic design to encourage app use:

People are going to want to look at something that looks kind of like nice and fancy...there is an importance to the aesthetic appeal of it.

Therapist 9

For me personally, I wouldn’t use this app if it didn’t look good to me.

Client 12

While they acknowledged that the app was a low-fidelity wireframe, they expressed that the visual design of the app was not on par with current market standards. The clients expressed a similar sentiment, expressing the need for the design to be adapted for the popular market:

It doesn’t look modern. It doesn’t look like today’s apps. It looks like...the beginnings of the internet.

Client 13

Some therapists appreciated what they described as a low-attention and boring design:

I see the value in a break from, you know being bombarded with color and moving stuff.

Therapist 3

By contrast, the clients expressed a desire for a more attention-grabbing, colorful design (client 8).

A recommendation to enhance the app’s aesthetic focused on reducing the amount of text. For instance, therapists suggested simplifying the exercise titles to make them less wordy and using images to complement the list of exercises and metaphors (therapists 1 and 8). The clients echoed this idea, preferring symbols to represent words (eg, bookmarks and learn more):

You don’t need all those words like we have symbols...And you know that pressing that [symbol] will add the exercise to the bookmarks. And you know that an eye in a circle means you can get more information for example.

Client 10

Both the therapists and the clients shared suggestions to improve ACTaide’s color palette. Drawing inspiration from other mental health apps they have experience with, some therapists recommended incorporating background colors, such as green and blue, into the app (therapist 1). The clients specifically generally requested greater contrast in the colors, feeling that the app was “plain” (client 1). The therapists leaned toward wanting nature-inspired colors in the color palette, a suggestion that echoed client feedback favoring earthy tones for a calming effect (client 13):

I would like to put nature-like colors...the browns, the greens, mainly blue sky.

Therapist 4

Similarly, clients provided suggestions to improve the appearance of the slider feature by incorporating colors to visually represent changes in their feelings.

Other specific suggestions made by both the therapists and the clients were discussed to rectify certain elements of the app, such as the size and location of the icons, with the goal of enhancing the app’s appeal:

The icons at the bottom...feel like they’re a little too big for the circles.

Client 2

**Figure 2:**
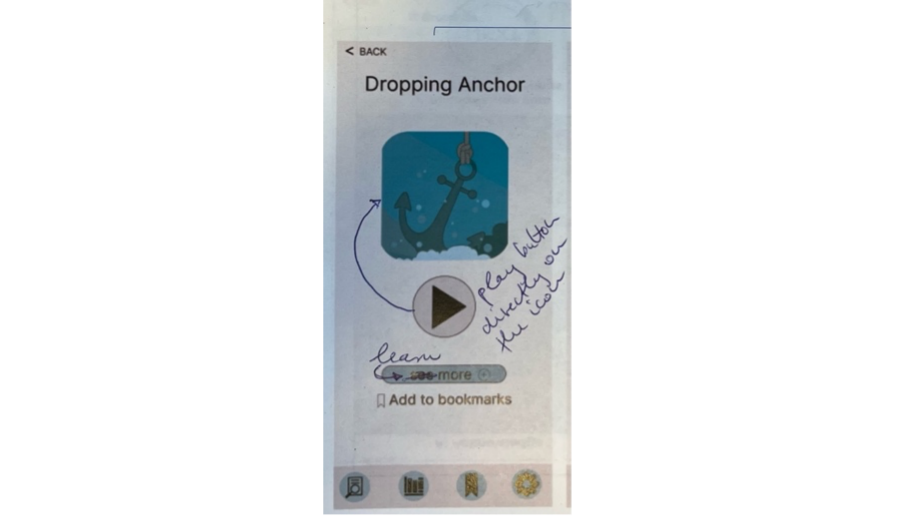
Implementation ideas for an exercise start page, generated by the clients in the fourth focus group. Suggestions focused on enhancing visual appeal and improving clarity.

The clients further found some pages to be fragmented and preferred simplicity. For example, they recommended merging the play button with the cover image of the exercise to create a more streamlined visual (Figure 2):

Having the play button underneath the actual icon, it’s just, it all looks very, very fragmented and kinda clunky and kinda busy.

Client 10

Finally, some clients noted that our aesthetic seemed to more closely resemble a web page and suggested ensuring it remained consistent with typical app designs*:*

The landing page looks like an Internet website, whereas the other pages look like an app.

Client 13

### Ease of Use

Both the clients and the therapists offered their feedback on the intuitiveness and simplicity of the interface. Consensus was reached about the app being user-friendly, with most agreeing that the app seemed simple and easy to use. Many therapists made comparisons to their favorite mental health apps, noting that their simplicity and ease of use were what stood out:

It looks fairly simple and user-friendly.

Therapist 1

It’s easy...it’s not complicated.

Client 3

The clients and the therapists also agreed that there were often too many steps or clicks involved, which could overcomplicate things. For example, the therapists felt that there were too many steps (eg, a distress scale and breathing prompt) to complete before one could start practicing an exercise:

There seemed to be a lot of pages in between saying that you wanted to do the exercise and actually getting into the exercise.

Therapist 1

Similarly, the clients felt that having a home screen or a pop-up screen to learn more about a given exercise was unnecessary, with 1 noting that “it’s a lot of clicking” (client 10).

Both the therapists and the clients emphasized the importance of intuitive labeling across the app to ensure ease of use. For example, the therapists expressed that an overly minimalist design could pose challenges for some clients with various aptitudes in technology. For example, an arrow was not considered explicit enough for one to understand how to exit a given wireframe. The clients had similar concerns with the language of various labels, such as *all skills*, *snooze*, *see more*, and *bookmarks*, which they reported were not intuitive. For example, the term *bookmarks* was considered confusing, as it is commonly associated with internet browsers rather than mobile apps (client 13). Alternative suggestions were agreed upon, such as *exercises*, *learn more*, and *favorites*:

It’s not clear to me what would happen if I click on that [referring to the see more button]. Maybe learn more.

Client 6

Similarly, the clients also reported that certain icons were not intuitive, such as the play button to start an exercise. They believed that this was not intuitive, as it primed them to expect a video (client 4). The bookmark icon and the library icon were also not rated as intuitive, with suggestions made to align them with their new labels (eg, a heart to represent favorites and a dumbbell to represent exercises).

While the clients acknowledged the value of various app features, such as reminders and a journal, they reported that such features should be more visible in the app. For example, they proposed moving such features into the navigation bar for easy access, as the previous ways of accessing such functions were not intuitive.

Finally, clients uniquely expressed a desire for increased app guidance generally. For example, they requested an optional tutorial and a help button to provide technological support to ensure that the app was user-friendly:

[When you] download a new app, you have...a tutorial that [says] welcome, [and] then takes us here [referring to the list of exercises].

Client 6

### Customization and Personalization

Both the therapists and the clients underscored the need to incorporate more options for customization and personalization of the app and its features to ensure that it accurately reflects the clients’ realities and needs. For instance, the therapists suggested that the facial expressions in the graphics could be customized to the user’s felt emotions through an input where one could enter their primary emotion. Such a sentiment was echoed by the clients, who felt that such an option would allow the exercise to resonate better with their experience. A therapist stated the following:

The actual graphic could be changed based on the emotion they identified...[such as] anger, shame, sadness, fear, which is going to more or less encompass the majority of negative experience.

Therapist 9

The therapists also proposed that an optional journal feature, where a client could reflect upon a given exercise, may be useful for the clients:

There could be an additional page where the person is asked to reflect...have them provide feedback on how it was for them.

Therapist 6

The clients expressed a strong desire for this feature as well, believing that it would aid in the app feeling more personalized:

I like the idea of being able to take notes of my thoughts during this process somewhere in the app.

Client 7

Finally, the therapists and the clients alike appreciated the option for customized reminders for exercise practice. The therapists emphasized the importance of avoiding the default reminders, suggesting instead that the clients have control over the frequency of such notifications.

**Figure 3:**
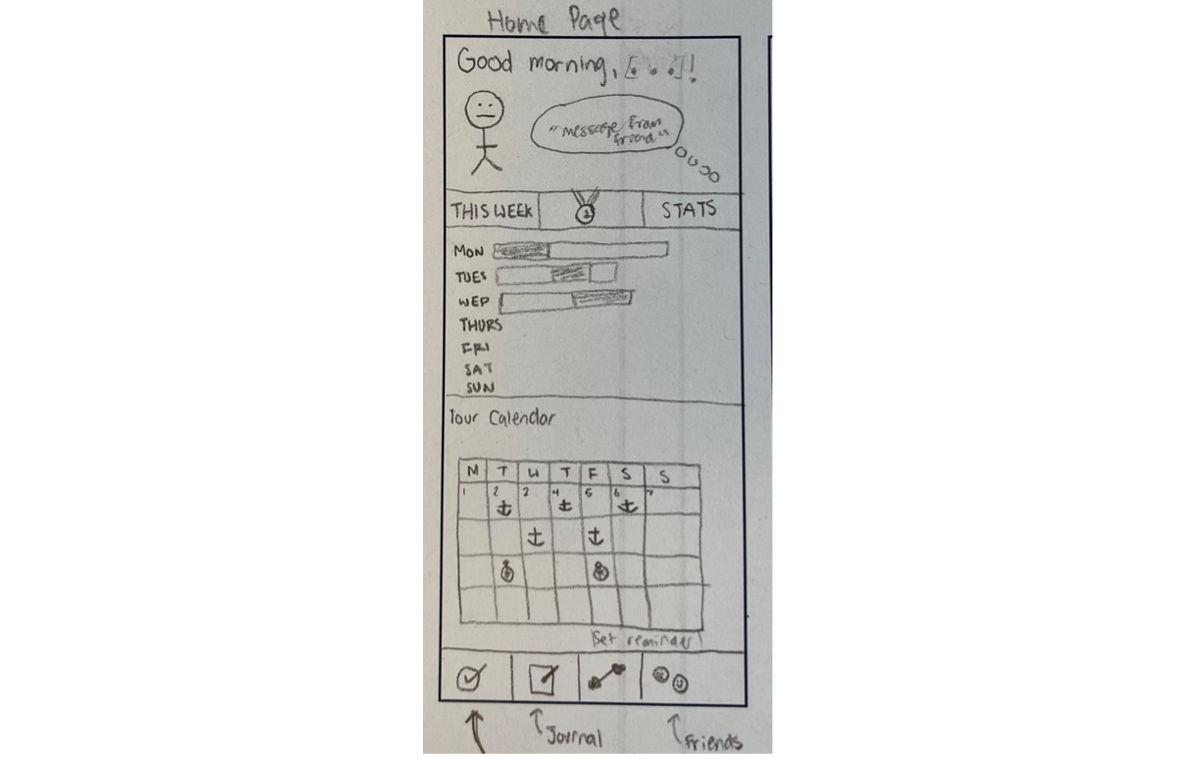
Implementation ideas for a home screen, generated by the clients in the fourth focus group. Suggestions emphasized personalization, gamification, and intuitive design.

The clients noted that the app had minimal opportunities for personalization, reporting that changes could be made to humanize the experience. They reported that small changes, such as being welcomed by the app with their name or with a personalized avatar, would make it feel less generic (Figure 3).

The clients and the therapists also requested further opportunities for customization throughout the app. For example, suggestions were made for the clients to customize the background color, stating that it would increase one’s desire to use the app (client 12). Furthermore, the therapists uniquely acknowledged that the clients may remember exercises by terms different from the formal labels used by their therapists. To rectify such discrepancies, they suggested an option for the clients to customize the label of the exercise based on their personal recollection of it (therapist 9).

### App Guidance

Both the therapists and the clients expressed a desire for the app to offer guidance regarding exercise selection. The therapists recommended incorporating more guidance in the form of assessments that can help identify the clients’ needs in a given moment. One would use the information from the assessment to guide them toward a specific exercise that targets the specific process or processes they are having the most difficulty with:

I wonder about maybe [adding] a question instead.... Something that is a bit more clear and is asking the client what they need in that moment? And then their answer to that will guide to the next part of the app.

Therapist 3

When such a feature was presented to the clients, they unanimously expressed appreciation for it, validating the therapists’ suggestions for assessments to guide exercise selection.

When the clients were shown the “get-recommendation” feature, a brief assessment to obtain a recommendation for an exercise to practice, they indicated a strong preference for completing the 6 items aligned with the hexaflex, as opposed to the 3 items aligned with the triflex. The clients reported favoring a 6-item questionnaire because it allowed greater specificity of recommendations and promoted self-reflection:

I wouldn’t mind answering six questions too if it gives me a better recommendation.

Client 12

In addition to desired guidance on exercise selection, the clients expressed consensus in valuing the “see-more” feature, wherein they could obtain a brief description of any given exercise and its purpose:

That’s very useful. I would definitely like to know what the exercise is about...before actually doing it.

Client 1

Some therapists similarly echoed the need for this feature, suggesting providing information on why a given exercise could be helpful or when one might want to try it (therapist 7). Most felt that very brief and optional preparatory psychoeducation could be helpful for clients:

Having some psychoeducation worked in...to remind people...why try this...would be really helpful.

Therapist 9

### App Engagement and Gamification

The therapists and the clients alike expressed a need to enhance app engagement. The therapists expressed mixed views about the use of gamification. Some felt that gamification could undermine intrinsic motivation:

[Gamification] removes kind of deliberate, kind of willful agency.

Therapist 3

Others appreciated the use of gamification features, such as progress tracking, that could reinforce app use, comparing it to other apps on the market that integrate such features. For example, 1 therapist commented that integrating a progress tracker would allow people to think, “hey, I’m building up this habit and feeling good about it” (participant 9).

By contrast, the clients reached a consensus that gamification elements would be their preferred way to increase app engagement. The clients emphasized their desire for similar progress tracking features to promote a sense of reward and achievement:

There’s no sense of achievement in the app.

Client 4

Something that help us to see, maybe a graphic,...what is the progress made by this app.

Client 5

Some suggested integrating goal-setting features for exercise practice:

You set a goal...right after your last therapy session, [such as] “I want to do my exercise like two times a week”..., then it shows your progress on your goals.

Client 9

Furthermore, both the clients and the therapists expressed concerns that the clients may bypass the exercise content by mindlessly skipping through the steps in the annotated image sequences without fully engaging with them. To address this concern, both the clients and the therapists suggested embedding questions and forced-choice prompts within the steps to require user input, such that the exercises are more interactive:

If you just have the app as is with images, it’s so easy to just press next, next, next without actually doing the steps. There has to be some sort of input because then otherwise I wouldn’t even want to go on the app to do it because...[I can do it] on my own.

Client 9

The clients proposed that greater interactivity could be achieved by incorporating sound and animations into the image sequences. They also expressed that an option for text to be read aloud might enhance their desire to engage with the exercises.

Finally, the clients considered the utility of a social component to encourage app engagement, such as the ability to connect with others by sharing their progress or sending words of encouragement:

I wouldn’t want people that I’m friends with necessarily knowing which exercises are completed or not.... But just some sort of indication that they have also been working on their exercises.

Client 9

The participants drew comparisons to other apps they were familiar with that use similar strategies. While some expressed interest, others noted that psychotherapy is a personal activity and were hesitant about such a feature (participant 9).

### Therapist Involvement

The potential for therapist involvement through a designated interface that would allow the therapists to assign exercises and monitor progress was a significant focus across the therapist focus groups. Such interaction was queried in the client focus groups but was not elaborated upon as a topic of discussion. Some therapists were initially interested in an optional shared data feature that would allow them to access patient data to monitor user engagement. Through further discussion, the therapists identified that such a feature was not likely to be useful, given the additional time necessary to examine the data:

It might be useful to know how much time they spent on the app during the week and...where they spent the most time. But...I don’t know that I would be pulling up the data.

Therapist 1

Further clinical concerns were echoed by most therapists, who felt that such a mechanism may negatively impact patient autonomy and motivation:

I want them using it for their own reasons and not because they feel they’re being watched or that they’re accountable to someone.

Therapist 1

However, 1 therapist observed that encouraging a sense of accountability might be clinically useful for some clients who lack intrinsic motivation:

Being accountable to your therapist for actually doing the exercises, might be tolerated and actually clinically useful for these patients..... I think the ability for them to feel that they are accountable to a therapist might actually promote usage and psychological flexibility.

Therapist 3

Similarly, the therapists initially expressed interest in taking an active role by assigning exercises to clients using a therapist interface, rather than having all exercises available to the patients:

I really like the idea of having things sort of locked or unlocked...Therapists will have some sort of control over the order in which things are...opened up to the patient.

Therapist 1

Others did not see the need for restricting access to exercises and metaphors:

I am okay with my patients going further and attempting things without me having said we’re working on this thing and then coming back.

Therapist 2

Most therapists once again acknowledged the burden of such a task, sensing that it would further add to their workload.

### Accessibility

The therapists emphasized the importance of accessibility throughout the app design, a topic that was not a focus of the client focus groups. The therapists appreciated the use of annotated image sequences with minimal, nonacademic text for promoting accessibility across people with differing education levels:

It is accessible...for people who might have difficulties with reading or benefit from something that is more image-based.

Therapist 8

More specifically, 1 therapist mentioned that images could facilitate the comprehension of content for clients who are not fluent in English. Some therapists expressed a desire to adopt a more universally accessible app design. For instance, they were keen on including an auditory component for clients with visual impairments:

An accessibility feature that has an audio option [would be necessary]....if it’s someone who has any type of visual impairment, this app will be inaccessible to them.

Therapist 8

All the therapists recommended further improving accessibility by reducing academic jargon, such as removing hexaflex terminology from exercise categories visible to the clients.

They also suggested enhancing visual and tactile accessibility by incorporating options to adjust the font and button sizes.

### Clinical Concerns

Various clinically relevant concerns were raised across the therapist focus groups. More specifically, the therapists expressed their doubts regarding the clinical utility of certain ACTaide features and their alignment with ACT principles. For example, the distress rating scale garnered the most concern, as it was deemed to be inconsistent with clinical use and geared toward research purposes. Specifically, the distress rating scale was poorly received, given that the therapists felt that focusing on reducing distress conflicted with ACT’s goal of enhancing functionality rather than targeting symptom reduction:

But the goal is not to reduce distress...it’s to tolerate it...Reduced distress is sometimes a by-product, but it’s not the emphasis.

Therapist 1

One participant noted that it is illogical to expect a change in distress after a brief exercise, while another raised concerns that clients might feel disappointed if they do not perceive immediate changes:

Is it realistic to expect the change after a 3 to 5 minute exercise?

Therapist 5

By setting up expectations of change among clients, the therapists expressed that the distress scale might compel some of them to feign their levels of distress, rendering the assessment meaningless. Finally, 1 therapist noted a preference for clients to engage with the exercises beyond moments of distress, which the distress scale seems to discourage by implying that users are distressed before using the app (therapist 3).

Most therapists felt that the “breathe feature,” which prompted clients to take a deep breath before doing an exercise, was not clinically appropriate, as the anchor of attention on the breath is not universally clinically indicated (therapist 5). They suggested using the phrase “take a moment” instead, to make it more accessible for all clients (therapist 1).

Finally, while the therapists appreciated the functionality of the favorites feature, they expressed hesitancy toward the language, proposing an alternative, such as “bookmarks.” The term “favorites” was believed to encourage the practice of only easy exercises, thereby creating an environment for clients that promotes avoidance of more challenging exercises:

Some of the exercises that might be most helpful to people actually might be the ones that are their least favorites...They might really dislike them, and yet it’s still going to be good for them to do it. So, I think it almost sets up avoidance.

Therapist 9

## Discussion

### Principal Findings

This study explored therapists’ and clients’ feedback on a low-fidelity prototype of ACTaide, a mobile app designed to enhance between-session practice of ACT exercises. The participants provided valuable insights into their preferences and perceptions, which guided iterative refinements of the app’s wireframes. A total of 9 themes emerged, reflecting both shared and differing perspectives between the therapists and the clients. Both groups emphasized the value of an adjunctive app for between-session practice and highlighted the need for a user-friendly and intuitive design. They preferred a streamlined interface with high-quality visuals rather than text-heavy features, while also advocating for greater interactivity to encourage consistent use. Personalization and customization were seen as essential for meeting diverse user needs. The therapists offered additional professional insights, including suggestions for maintaining alignment with ACT principles, increasing accessibility, and clarifying the therapist’s role in app delivery. These findings provide actionable guidance for designing mobile apps that balance clinical goals with user preferences.

Usability emerged as a critical theme, with participants emphasizing the need for a streamlined, intuitive interface. According to the International Organization for Standardization, usability is commonly defined as “the degree to which a product or system can be used by specified users to achieve specified goals with effectiveness, efficiency, and satisfaction in a specified context of use” [90]. The factors that contribute to the usability of an app include the amount of effort required to learn and use the app [91]. This finding is consistent with perceptions of both consumers and mental health professionals, who highlight ease of use as one of the most valuable app features [92,93], with ACT therapists specifically identifying it as a key criterion they use for evaluating ACT apps [63]. Specific suggestions, such as reducing the number of clicks, simplifying navigation, and providing onboarding tutorials, mirror well-established usability heuristics [94,95]. These recommendations were particularly salient among the clients, who noted that overly complex designs could detract from their willingness to engage with the app. Changes based on client feedback, such as incorporating intuitive icons, were iteratively made to further simplify the wireframes. Icons have been found to enhance app navigation and improve comprehension [96], provided they are familiar to users and unambiguous [91]. Similarly, suggestions were made to increase the visibility and location of certain app features, such as reminders and the journal. Older adults have identified such enhancements as crucial for improving the ease of use of apps [97]. Finally, the clients desired greater app guidance and requested an optional tutorial. This aligns with app development recommendations emphasizing that onboarding instructions should be provided for first-time users, regardless of how intuitive the app is perceived to be [61,98].

Consistent with prior research emphasizing the role of aesthetics in fostering digital engagement [99,100], both the therapists and the clients emphasized that a visually appealing app would be necessary to encourage app use. Research has shown that an app’s aesthetic design correlates with an app’s perceived usability [91]. The focus on aesthetic design is unsurprising, given that users tend to focus closely on aesthetics and visual interface elements during their initial experiences with apps [101]. Both the therapists and the clients focused on providing suggestions to enhance the app’s visual appeal. For example, the color palette was a focal concern, with participants requesting calming colors, such as blues and greens. Research has shown that blue and green tones with medium saturation and high brightness, a color scheme common to medical apps, evoke a sense of calmness, trust, and professionalism [102]. Given the influence of colors on emotions and visual appeal [100], such feedback was implemented into iterative wireframes.

The participants further highlighted the desire to customize many of the app features, such as the labels, reminders, images, and colors. Designing mental health apps for a wide audience can be achieved by incorporating opportunities for the app to be personalized to align with individual needs and preferences [61]. Customization has been identified in mental health apps as a factor positively affecting both app effectiveness and engagement [103,104]. However, it is not commonly implemented [105]. For example, the therapists acknowledged the value of reminders but emphasized the need for end users to customize their timing and frequency. This iterative change was appreciated by the clients in the subsequent focus groups, echoing the feedback given by users in other studies regarding customizable reminders [61,77,106]. Such customization strategies in mental health apps have been shown to foster end users’ sense of self-agency and self-determination, ultimately enhancing their engagement with apps [91].

Concerns and suggestions to improve overall app engagement emerged across focus groups, reflecting the widespread issue of low engagement faced by many mental health apps [58]. Gamification to promote engagement emerged as a particularly contentious topic, with the therapists expressing concern that gamified elements might undermine intrinsic motivation, while the clients viewed such features as necessary to maintain interest. Research on gamification in mental health apps has yielded mixed results, with some studies demonstrating its effectiveness in promoting app use, while others have found the opposite [107,108]. For example, a meta-analysis on apps for depression found no difference in engagement between gamified and nongamified apps [109]. Progress levels, a feature that shows how much progress a user has made toward a goal or task, have been found in >40 mental health apps [110]. The common use of this feature may explain why clients emphasized the inclusion of a similar feature, often making comparisons to apps they have used before. The therapists expressed concerns about incorporating gamification, noting that it may hinder intrinsic motivation. This aligns with critiques that argue gamifying technology relies on extrinsic motivators and positive reinforcement, such as overt rewards, to motivate user engagement [111]. Research has shown that gamification in health technologies is frequently implemented without accounting for its effects on intrinsic motivation, without clear objectives, and without understanding its mechanisms [110,112]. To reconcile these contrasting views on gamification given by the therapists and the clients, a compromise might involve subtle gamification strategies, such as progress tracking and badges for completed exercises, that support engagement without detracting from the therapeutic focus.

Both the therapists and the clients highlighted a need to incorporate interactive elements into ACTaide to promote engagement with the exercises. According to the taxonomy of user interactivity [113], which refers to the degree to which users can input and modify system outputs, the wireframes presented place ACTaide at a static level of interactivity, with limited to no user input of personal data. Incorporating the therapists’ and clients’ recommendations, such as adding questions, forced-choice prompts, and animations, would elevate ACTaide from a static to a basic level of interactivity within the taxonomy. These changes are important, given that interactivity features are known to enhance user engagement in digital interventions [113].

The therapists were particularly concerned with their level of involvement in the delivery of ACTaide, reporting mixed feelings about the potential inclusion of a therapist interface that would enable them to monitor user engagement and assign exercises to the clients. The discussion was characterized by an immediate interest in these features, followed by concerns about the feasibility of implementation in clinical practice. Most therapists acknowledged that it would undesirably add to their workload. Such opinions have been expressed among other health professionals, who emphasized that any new technology they adopt in their practice should clearly enhance efficiency, noting that their auxiliary tasks were already time consuming [93]. This finding aligns with the therapists’ reluctance to take on additional responsibilities. Similarly, a qualitative exploratory pilot study exploring therapists’ perspectives on blended therapy (eg, the combination of psychotherapy with digital components) highlighted similar concerns over workload as a barrier to implementation [114]. Therefore, the therapists noted that their involvement in the interface would be unnecessary for the app to achieve its intended purpose for the clients.

The therapists also emphasized the importance of accessibility, expressing appreciation for the app’s strengths while noting areas for improvement. ACTaide’s minimal use of language and emphasis on imagery were considered suitable for clients with lower literacy levels, who often encounter language barriers when accessing mental health technologies [115]. The therapists also suggested reducing academic language, which aligns with findings from a systematic review that found that participants engage better with interventions that are tailored to them, which could be achieved by limiting the use of jargon and technical language [116]. This reduction in academic jargon not only enhances user engagement but also simplifies language, a factor that end users have identified as important in mental health apps [101]. Other suggestions to improve accessibility were made, such as changing font size and button size and adding optional features to accommodate end users with disabilities. A cross-sectional study exploring 578 mental health apps in the marketplace found that accessibility features, such as adjustable text size and text-to-speech or speech-to-text abilities, were present in less than half of these apps [117]. Thus, the findings highlight the importance of prioritizing accessibility features in iterative app design.

An important contribution of this study is the therapists’ identification of features that may inadvertently conflict with ACT principles. For instance, the therapists raised concerns about the inclusion of a distress rating scale, arguing that it risked reinforcing symptom reduction as a primary goal, contrary to ACT’s emphasis on psychological flexibility [118]. The results from an Association for Contextual Behavioral Science survey revealed that practitioners rated consistency with ACT principles as the most important criterion for evaluating apps [63]. This feedback underscores the value of incorporating professional expertise in the co-design process to ensure that digital tools align with the theoretical foundations of their respective interventions. Following this feedback, the distress rating scale was removed from the app, demonstrating how iterative design can safeguard intervention fidelity. Furthermore, the therapists disliked the “favorites” label, as it was thought to implicitly promote experiential avoidance of more challenging exercises. The term “bookmarks” was thought to be more inclusive. However, the clients disliked the “bookmarks” label, as it was not considered to be intuitive, and preferred the term “favorites.” This is an example of how a user-centered iterative design process allows one to refine and adjust the app based on the evolving preferences and needs of various stakeholders, which may differ by group [119].

### Implications

The findings from this study provide actionable insights for refining ACTaide and underscore the value of user-centered, iterative design in mental health app development. The feedback obtained from this study will be used in the iterative design of ACTaide, ensuring alignment with end users’’ needs and preferences. New wireframes based on the identified themes will be translated into a high-fidelity functional prototype. To refine the intervention, the next step will involve beta testing a high-fidelity prototype to assess acceptability, engagement, and usability in real-world settings. Aligned with the Obesity-Related Behavioral Intervention Trials model for designing behavioral interventions, further data will need to be obtained through a proof-of-concept study, pilot testing, and then a feasibility pilot in preparation for an efficacy trial [120]. A longitudinal efficacy trial could then evaluate the app’s impact on homework adherence and psychological flexibility, addressing a critical gap in the literature. Experimental studies might also benefit from testing the format of exercise delivery (ie, annotated image sequences) to assess the impact on comprehension and retention. The facilitators and barriers to widespread adoption of ACTaide may also be explored during implementation, alongside effectiveness in real-world settings.

The incorporation of the perspectives of multiple stakeholders can potentially promote the acceptability of the tool while enhancing uptake and retention [103,121]. Importantly, involving health professionals alongside end users in the design of mental health apps may boost confidence in the final product [61,122]. Research has found that endorsement of patient-facing technologies by mental health providers is associated with greater interest from end users [123], emphasizing the credibility of an adjunctive tool that has been co-designed with mental health professionals.

### Strengths and Limitations

This study has several strengths, notably its use of focus groups with a user-centered co-design framework, which facilitated in-depth discussions and provided a platform for eliciting feedback on a low-fidelity wireframe prototype of ACTaide from distinct stakeholder groups—the therapists and clients. Another strength lies in the diversity achieved within the client focus groups, supported by quota sampling across ethnicities, which largely reflected the demographics of Montreal [124]. This sampling approach allowed representation of a wide spectrum of client experiences, though only 1 older adult client participated, which likely mirrors the age distribution in psychotherapy clientele [125]. While the therapist focus groups showed less diversity, this likely reflects workforce demographics; in Canada, for example, <25% of psychologists are men [126] and >75% are aged between 30 and 59 years [127]. The iterative nature of focus group discussions also allowed the research team to refine wireframes based on real-time participant feedback, promoting a user-centered approach to app development. The geographical restriction to participants who resided in or were able to commute to Montreal, combined with the requirement for English proficiency, limits the generalization of findings to other regions or non–English-speaking populations.

### Conclusions

This study illustrates the feasibility and value of involving both users and professionals in the design of an adjunctive therapy app at the first stage of the process, aligned with the user-centered design approach [75]. The key themes identified were the value of ACTaide, aesthetic design preferences, ease of use, customization and personalization, app guidance, app engagement and gamification, therapist involvement, accessibility, and clinical concerns. By integrating feedback from both the therapists and clients, the app may address long-standing challenges of homework engagement through a user-friendly solution, the value of which was recognized by all stakeholders consulted. Through the integration of feedback from end users, ACTaide has the potential to address barriers to between-session practice, enhancing therapeutic outcomes and bridging the gap between therapy sessions in a creative, innovative, and scalable way.

## Appendix

Multimedia Appendix 1 Iteratively designed low-fidelity wireframes.

Multimedia Appendix 2 Summary of the design changes.

Multimedia Appendix 3 Semistructured interview guide.

## Abbreviations

ACT: acceptance and commitment therapy

## Data Availability

Data sharing is not applicable to this article. The datasets generated or analyzed during this study (ie, the audio recordings of the focus group sessions and the corresponding transcripts) are not publicly available because of confidentiality concerns and ethical restrictions imposed by our institutional review board. In addition, deidentified transcripts cannot be shared because not all participants provided consent for their deidentified data to be used in future, unspecified research. Moreover, given the inherently collective nature of focus group discussions, it is not feasible to disaggregate participant contributions.
