# Supplementary material for: Therapy Companion Mobile App for Acceptance and Commitment Therapy Exercises (ACTaide): Therapist and Client Co-Design Study
Source: JMIR Form Res. 2025 Jul 24;9:e69532. doi: 10.2196/69532 (PMC12332461; doi:10.2196/69532)
Supplement: Multimedia Appendix 1 [file formative_v9i1e69532_app1.docx]

**Therapist Focus Group 1**


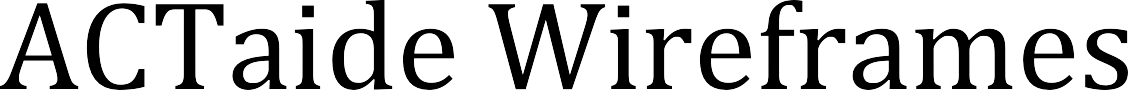


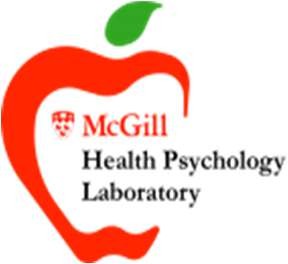


Home Page

Locked Exercise Page

Exercise Page (Overview)


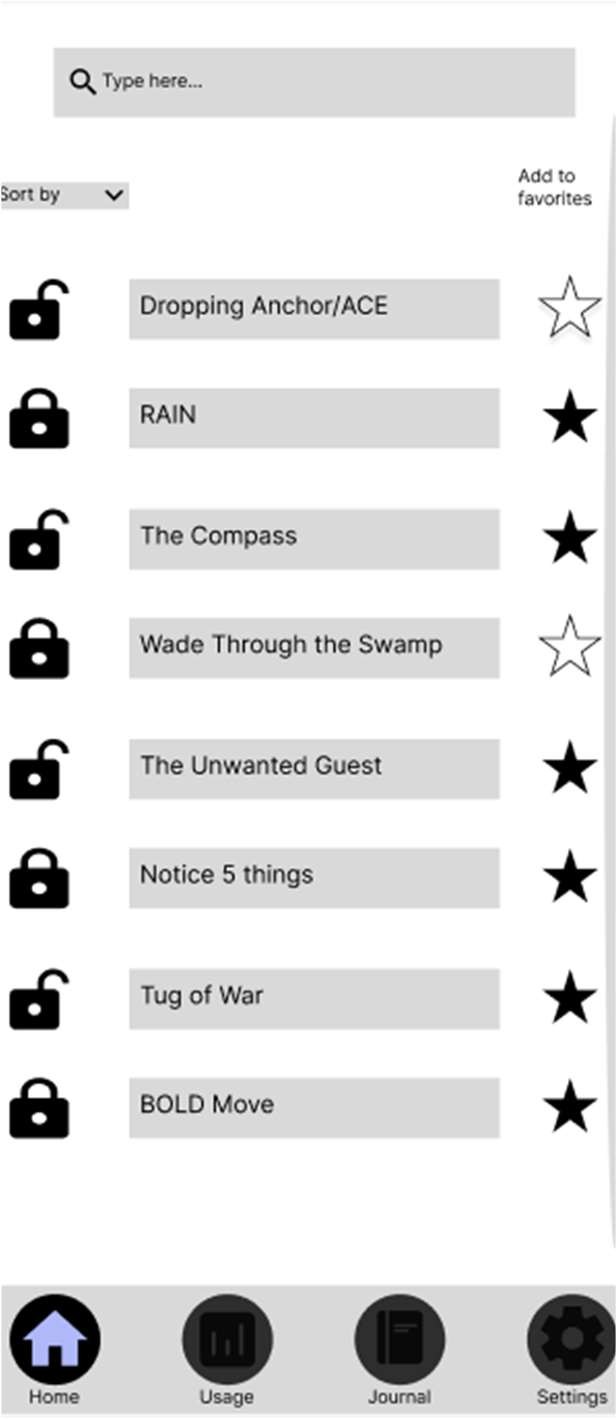

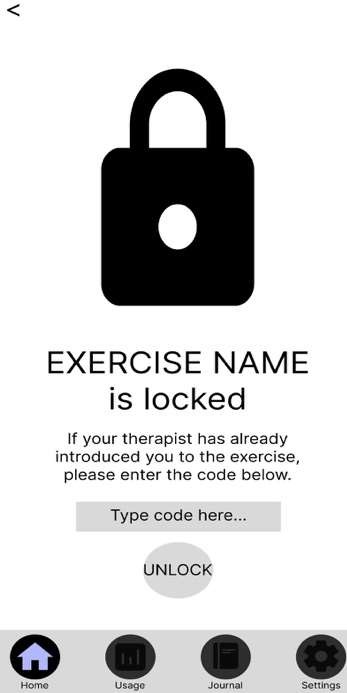

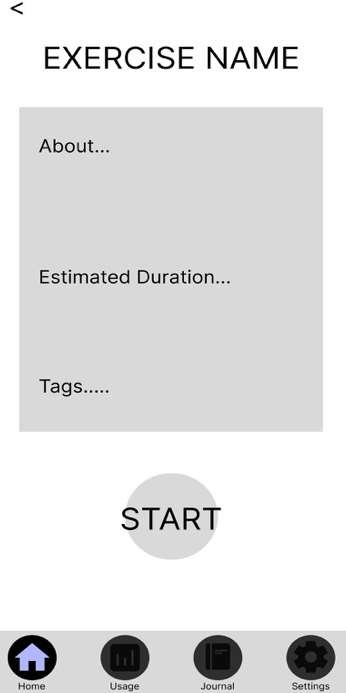


Distress Rating Scale (Post-Exercise)

Distress Rating Scale (Pre-Exercise)

Breathe Page (Pre-Exercise)

Settings Page


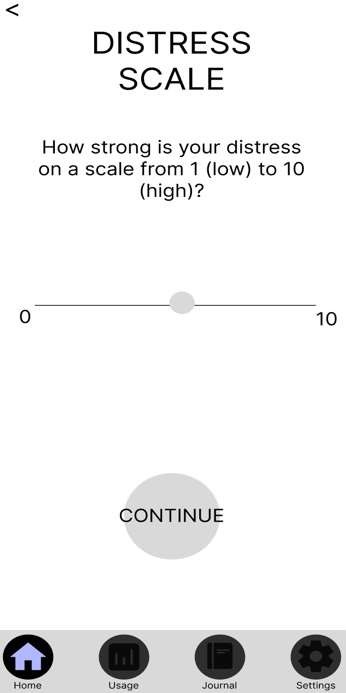

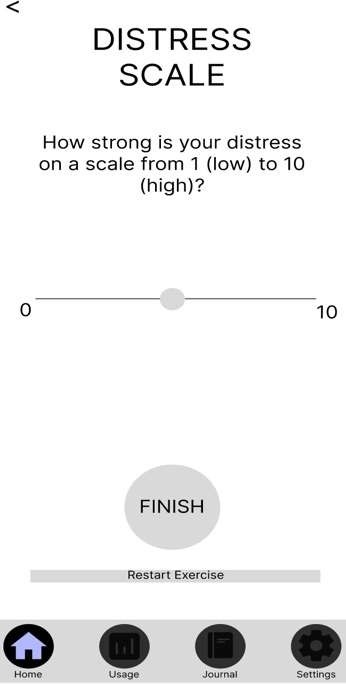

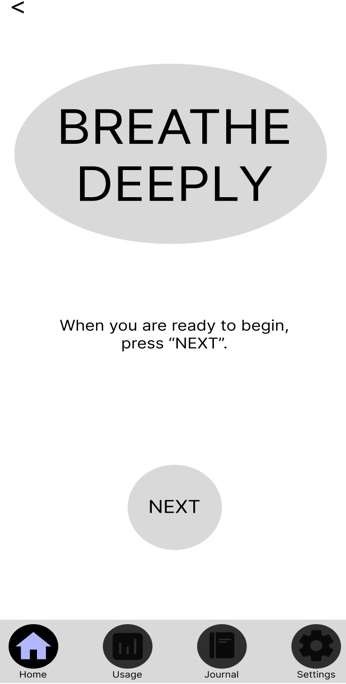

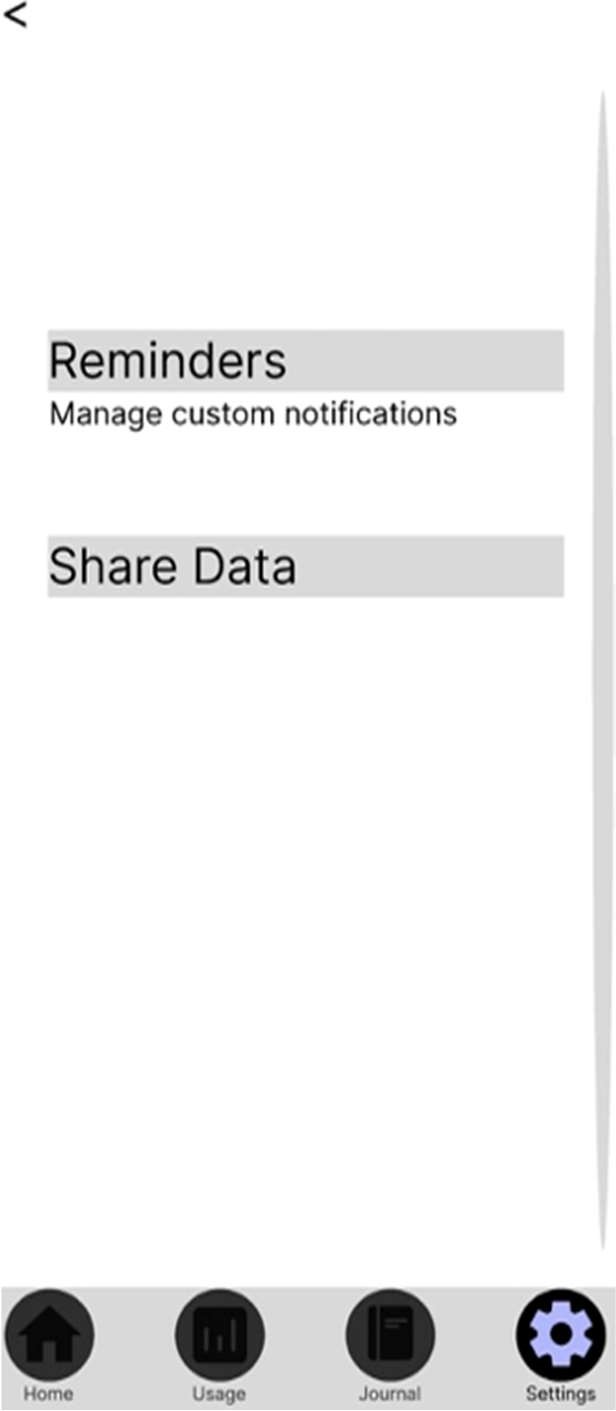


Annotated Image Sequence Pages: Dropping Anchor


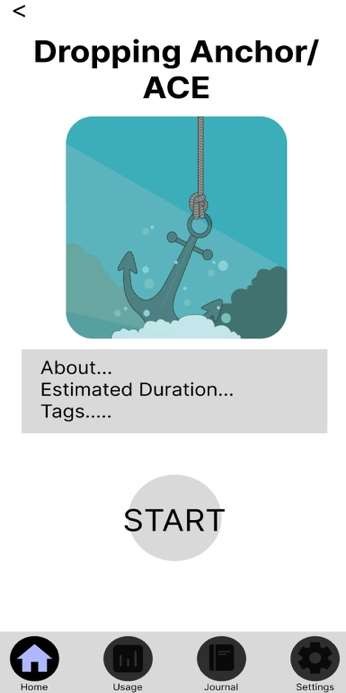

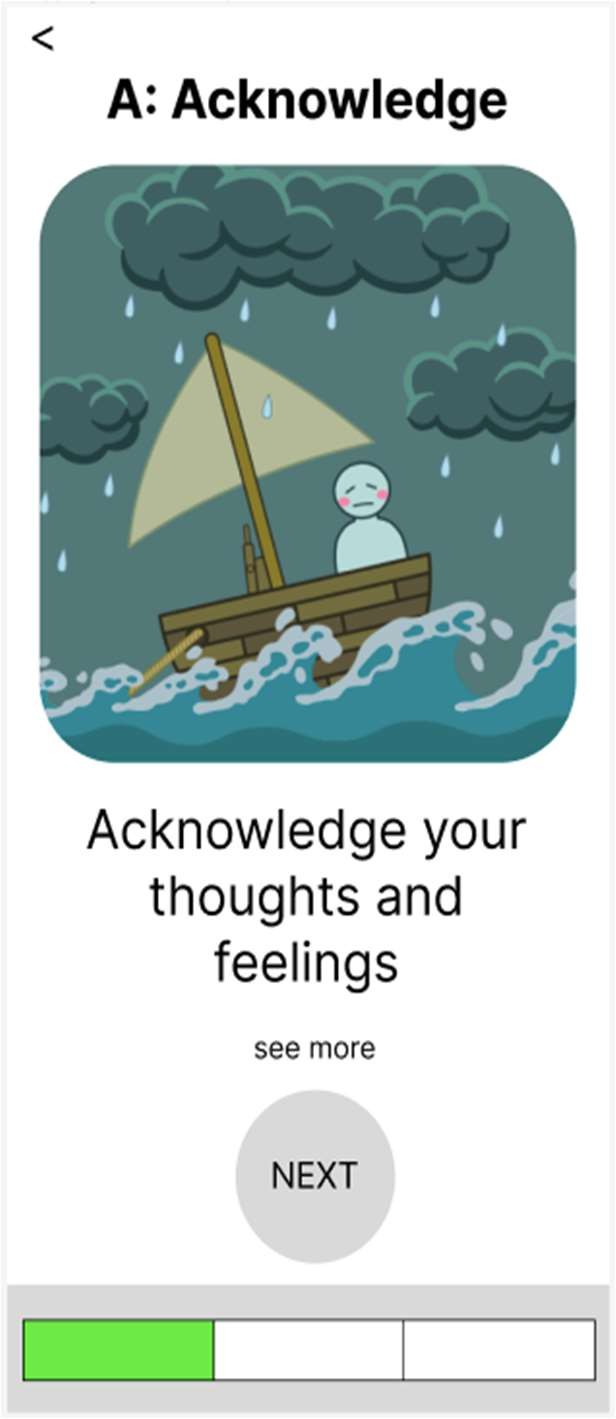

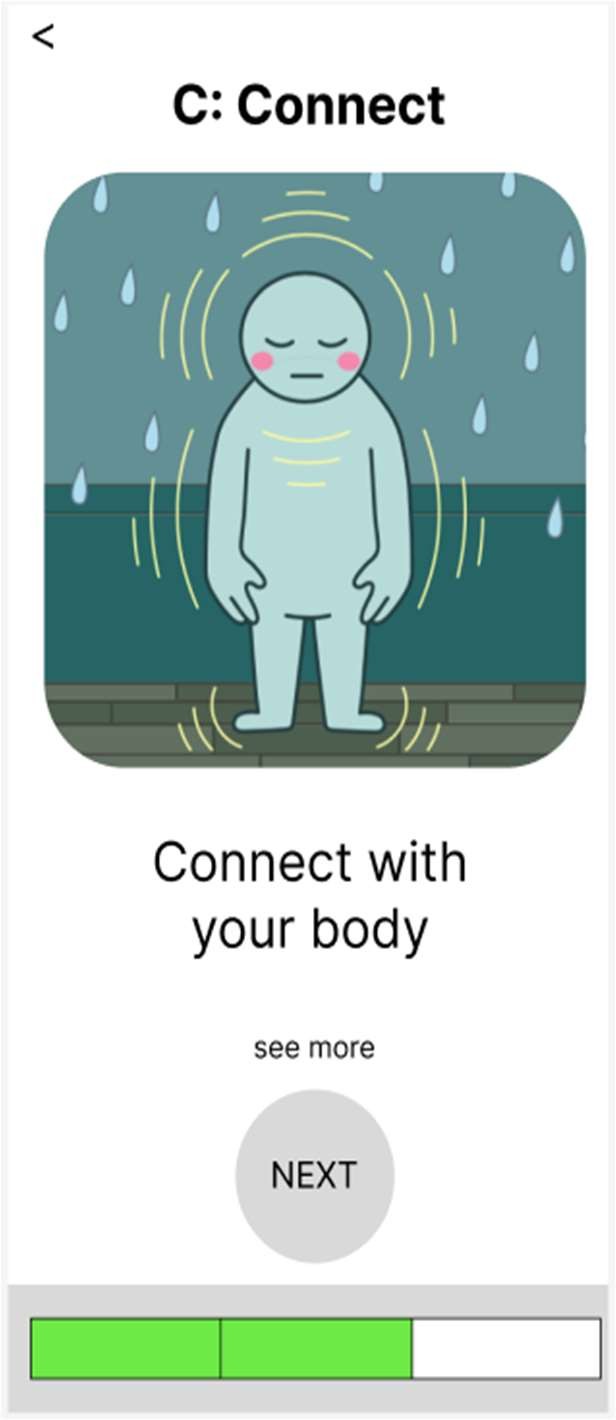

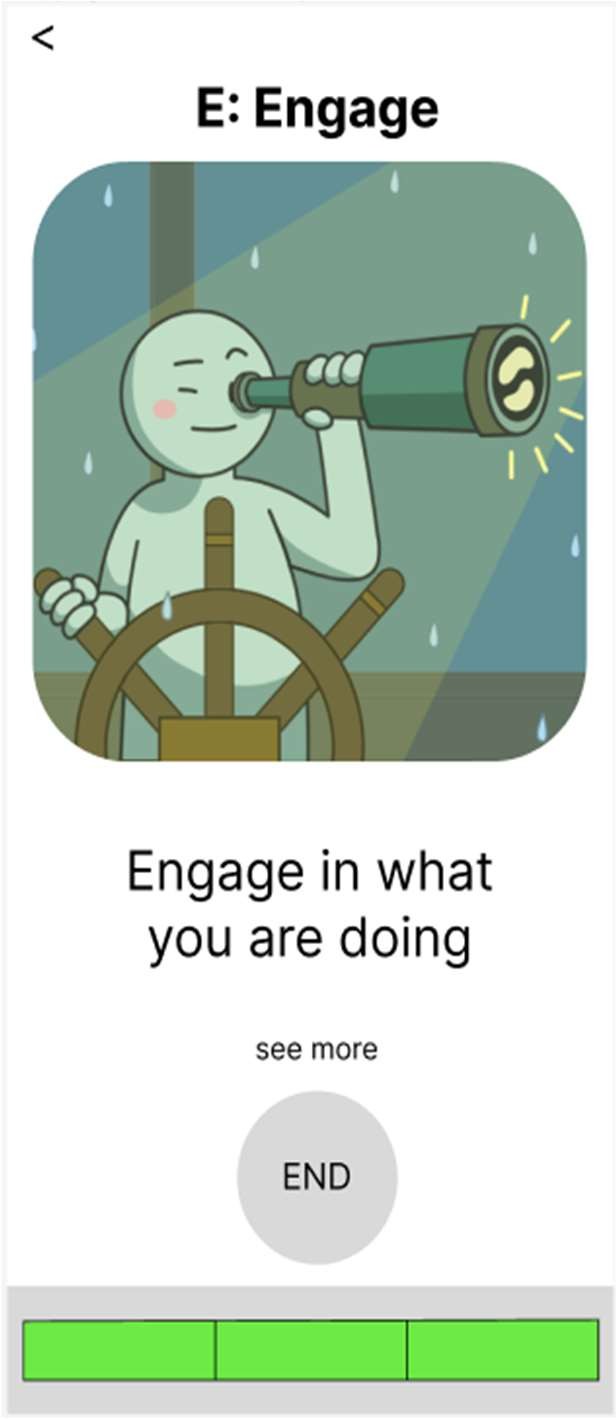


Annotated Image Sequence Pages: RAIN


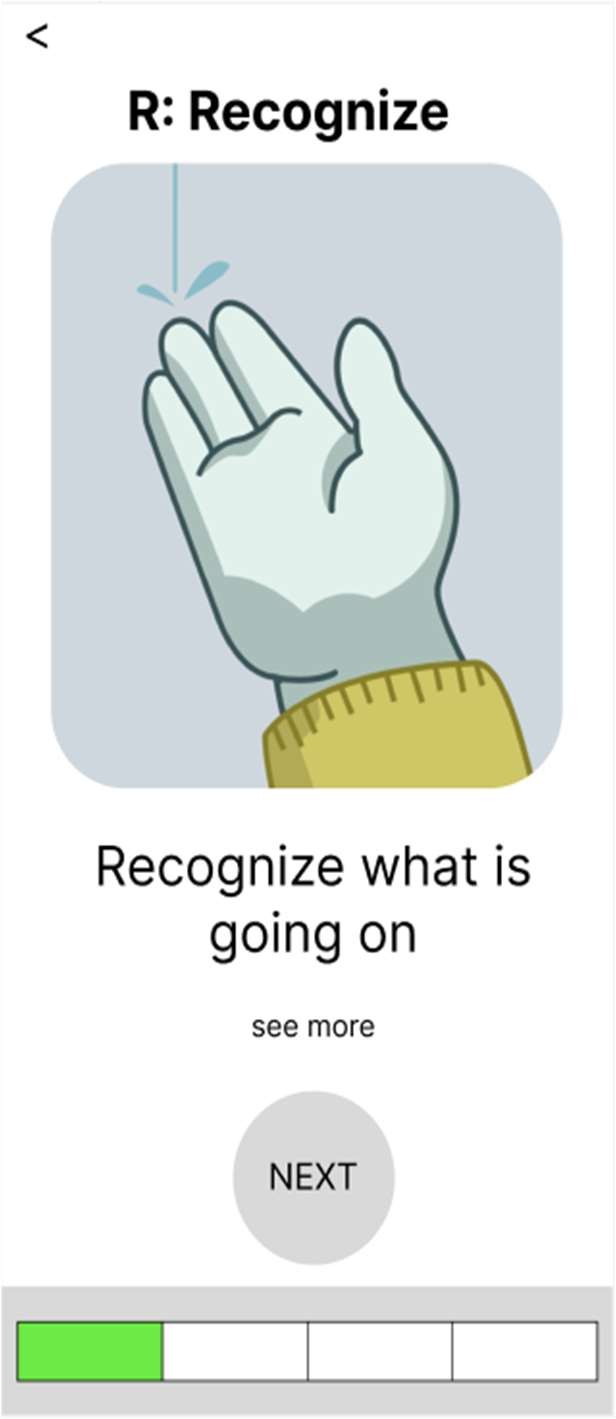

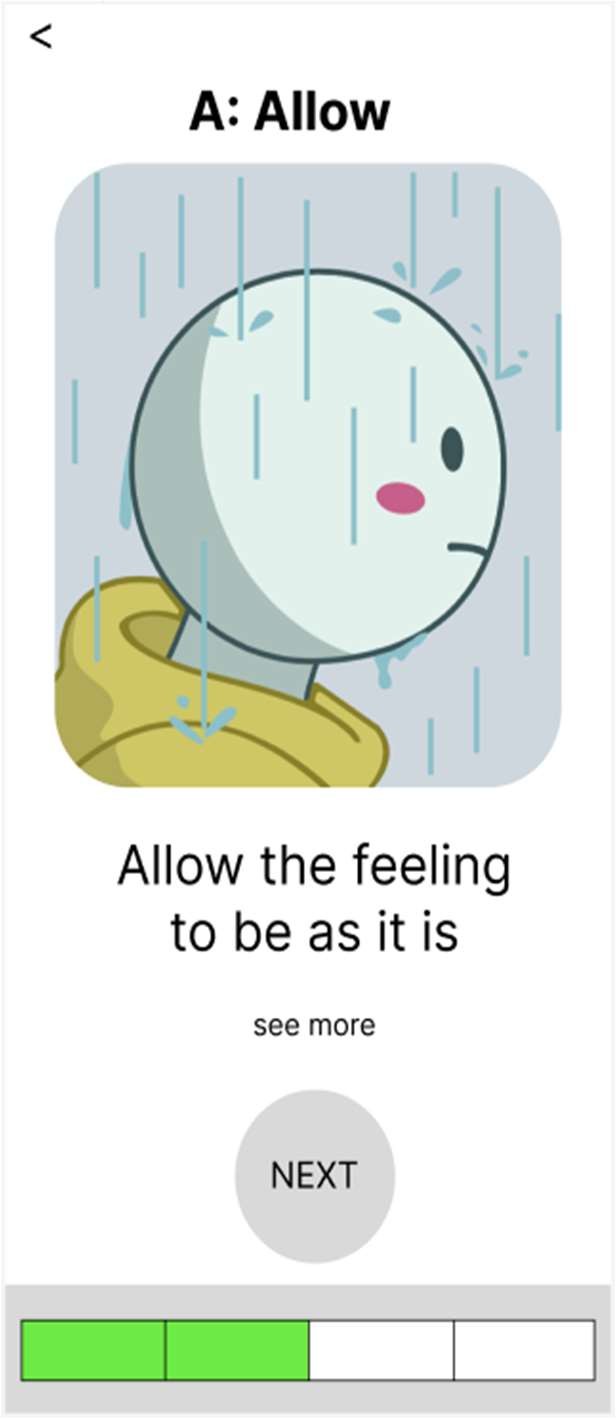

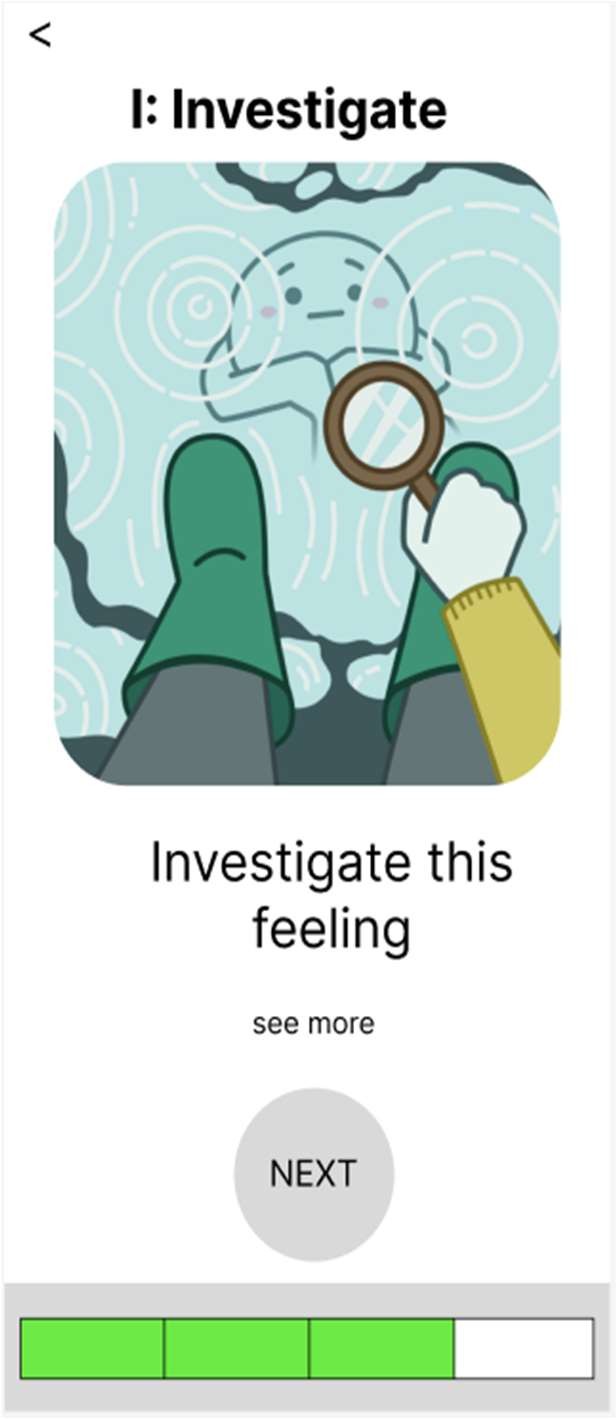

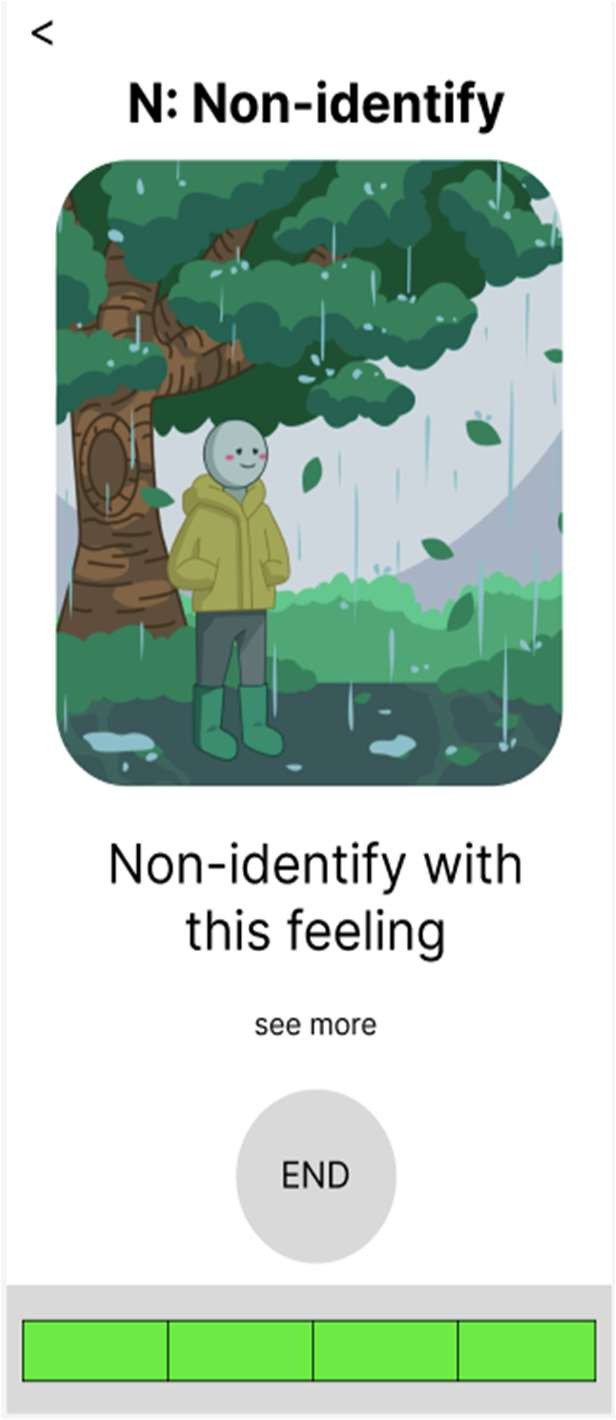


Annotated Image Sequence Pages: Compass


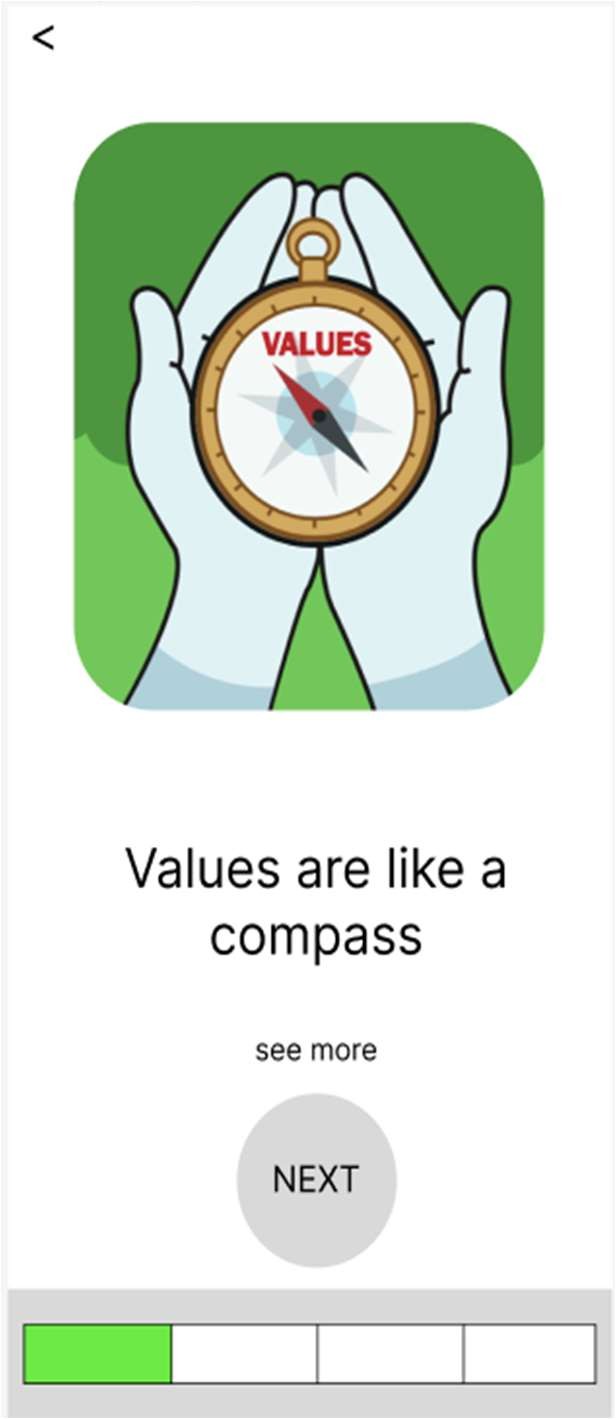

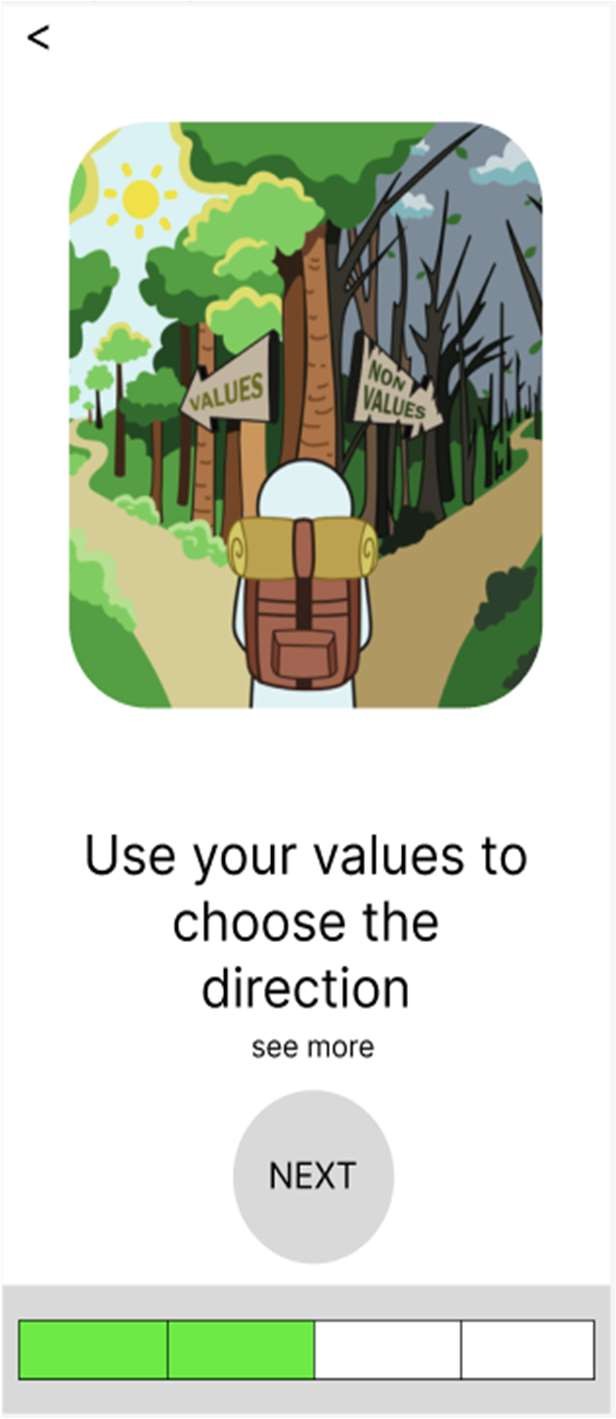

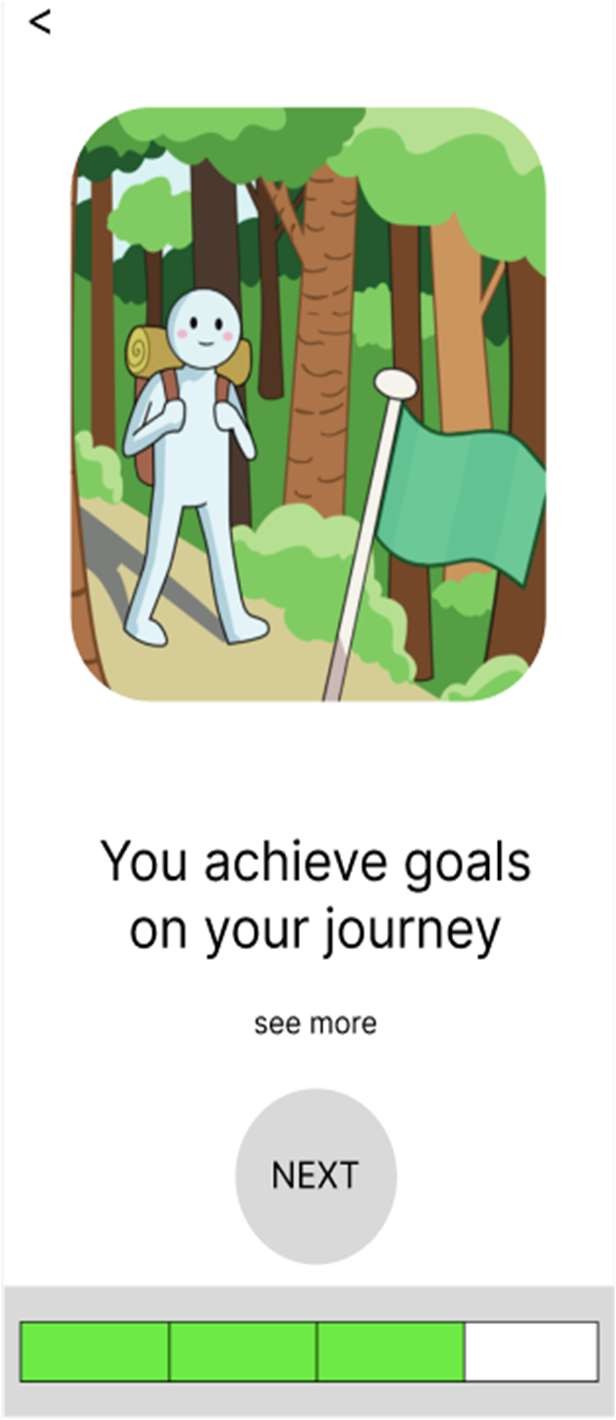

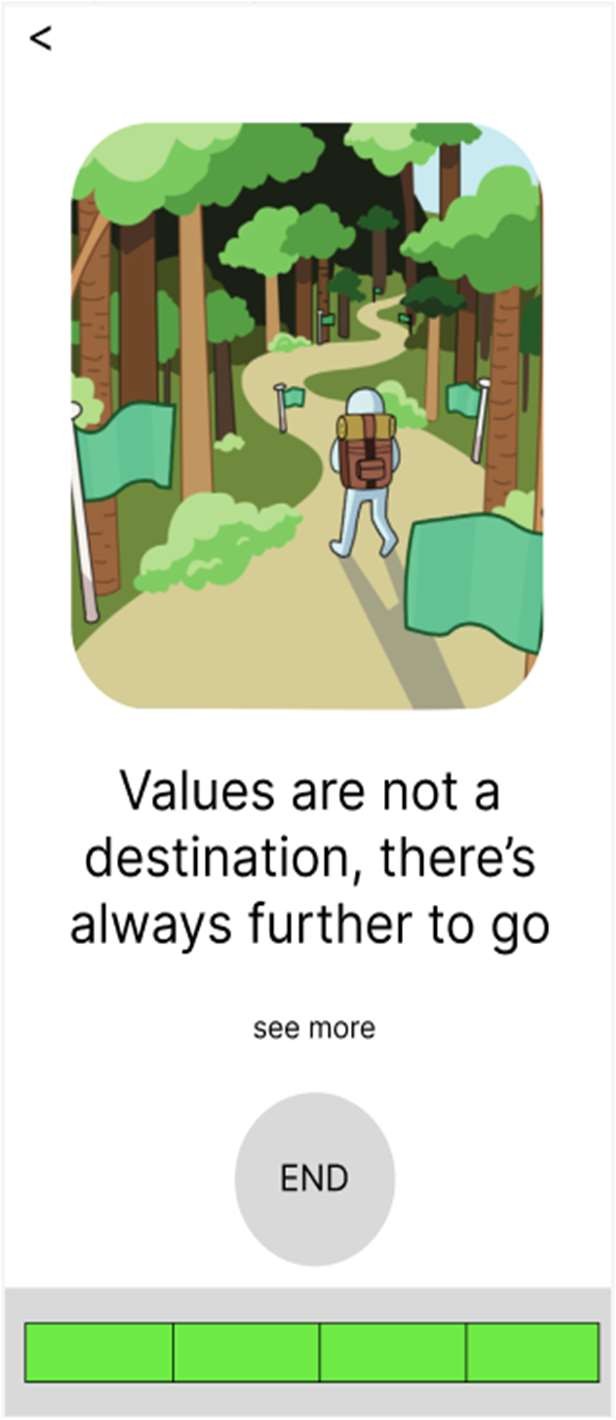


**Therapist Focus Group 2**

# ACTaide Wireframes


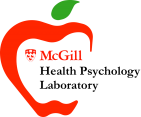


List of Exercises Page: Open Up

Home Page

List of Exercises Page:

Be Present

List of Exercises Page:

Do What Matters


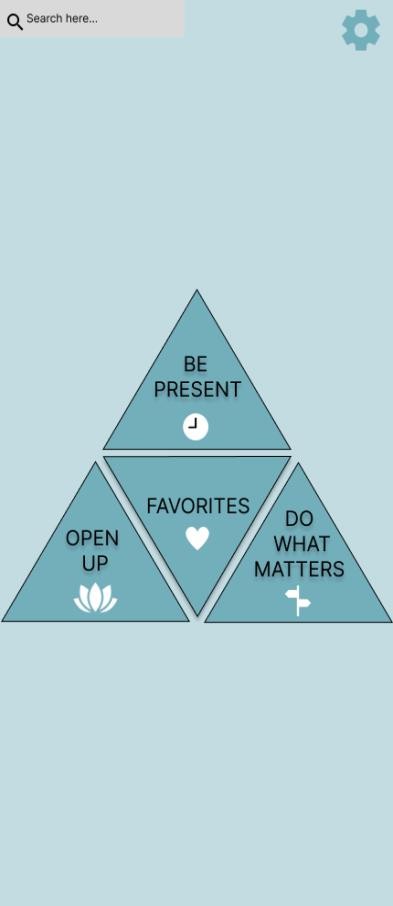

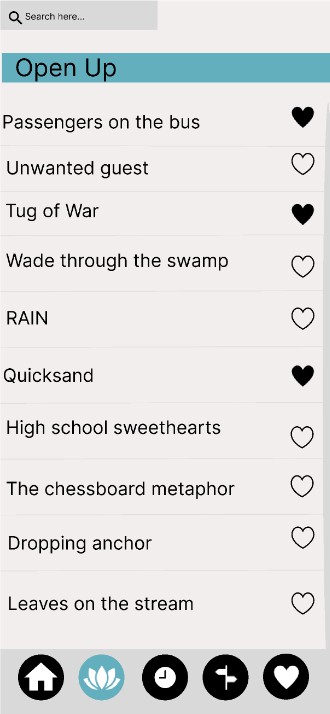

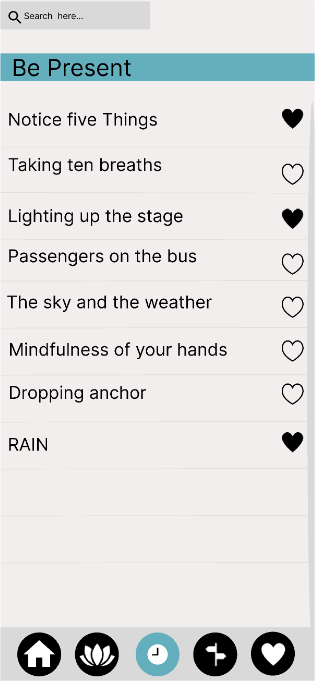

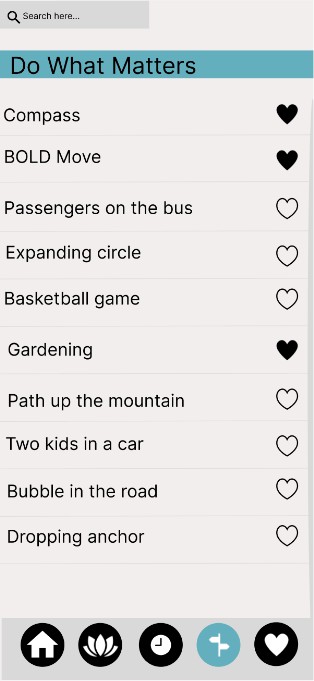


List of Favorite Exercises Page Start Exercise Page See More Page Take a Moment Page


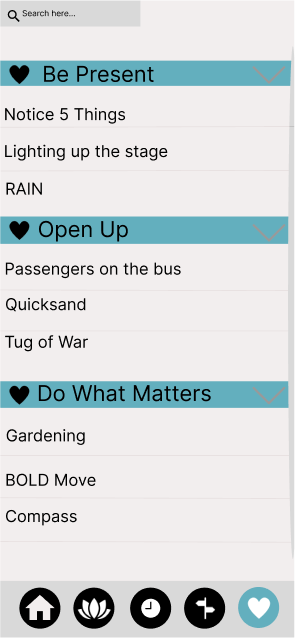

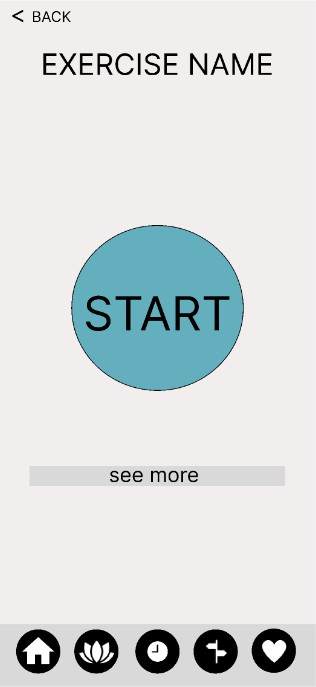

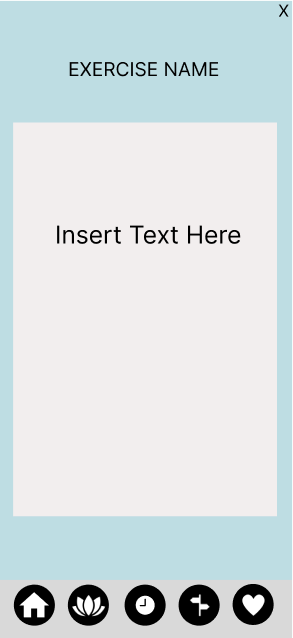

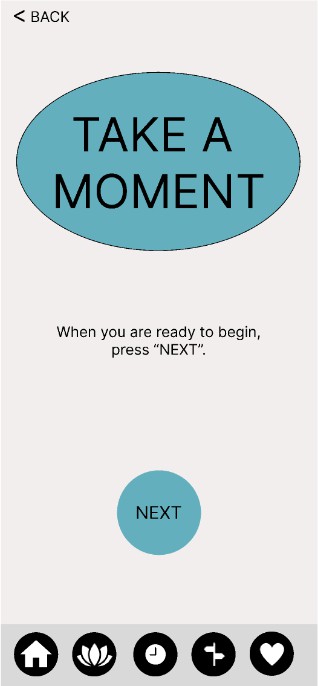


Annotated Image Sequence Pages: Dropping Anchor


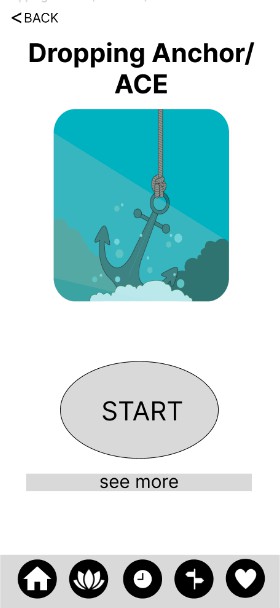

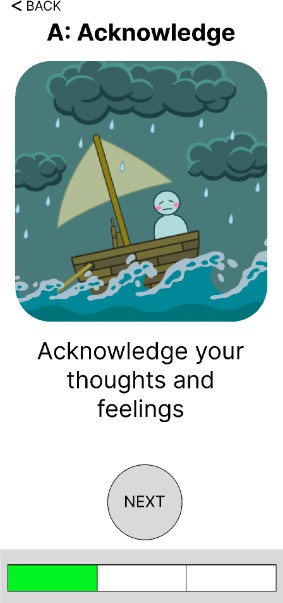

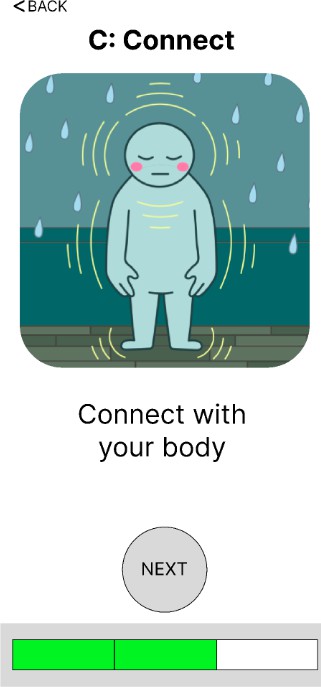

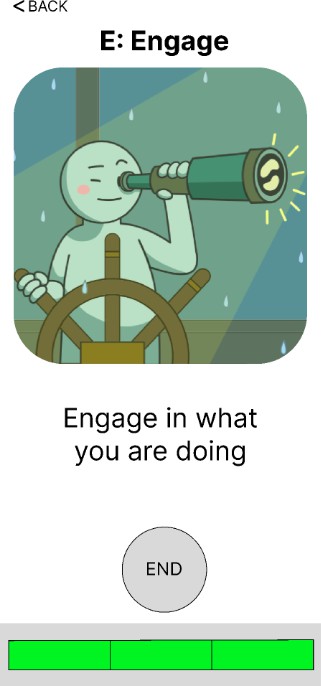


Annotated Image Sequence Pages: RAIN


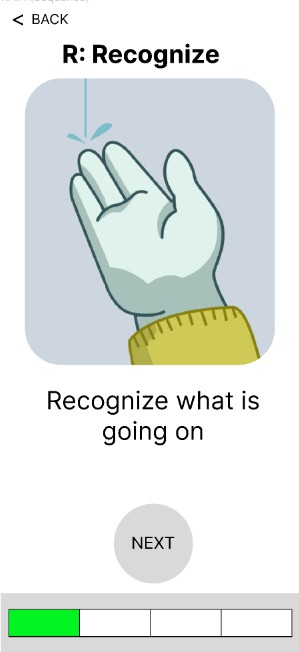

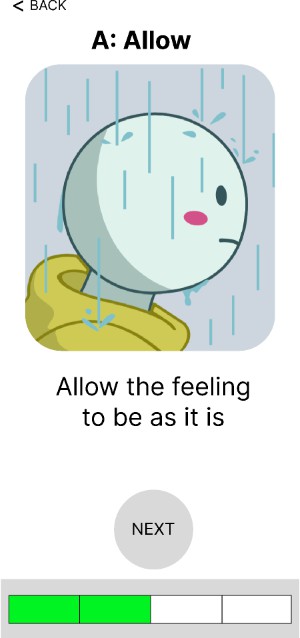

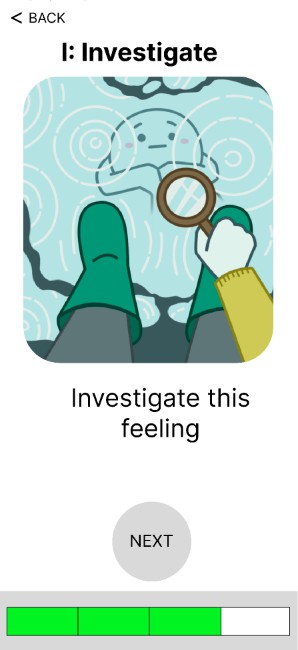

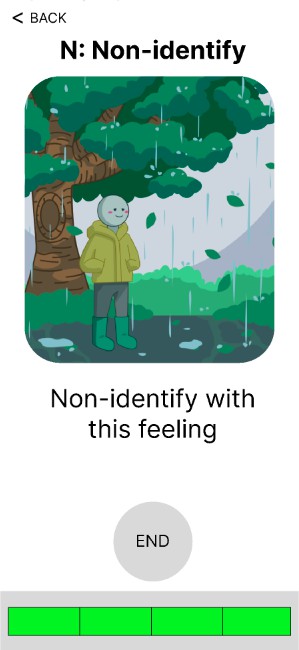


Annotated Image Sequence Pages: Compass


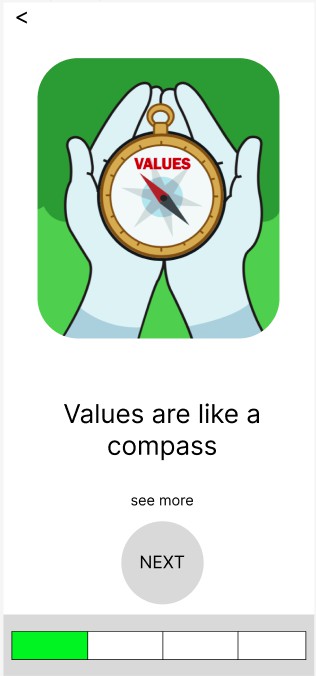

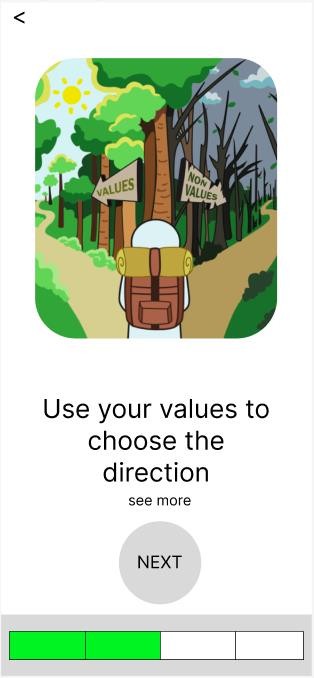

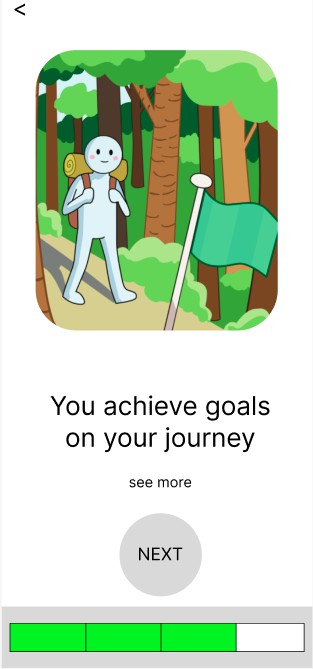

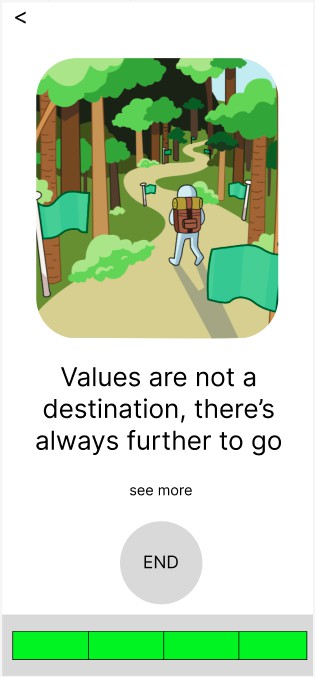


Settings Page Reminders Page Add Reminders Page Edit Reminders Page


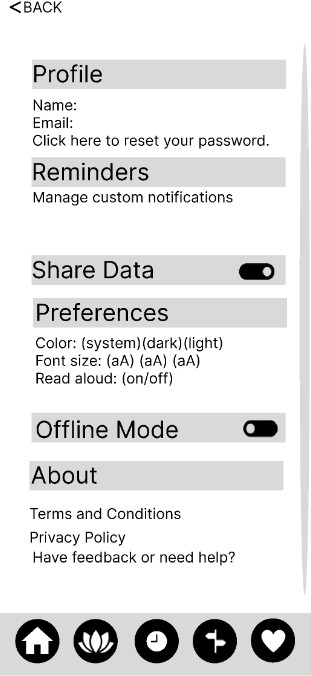

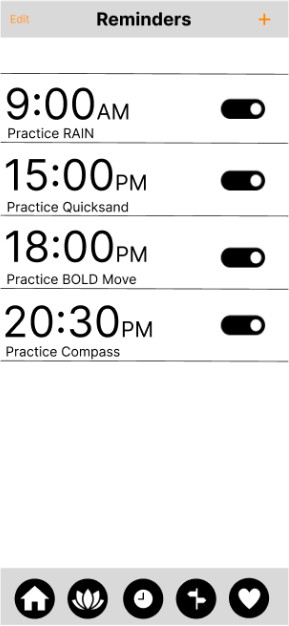

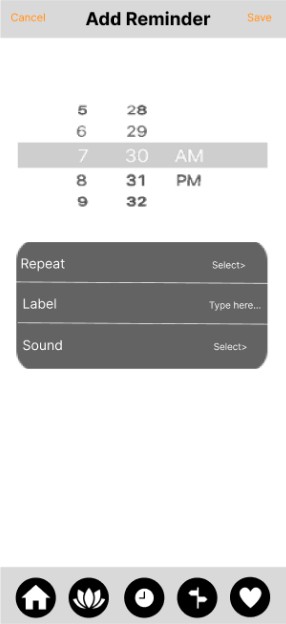

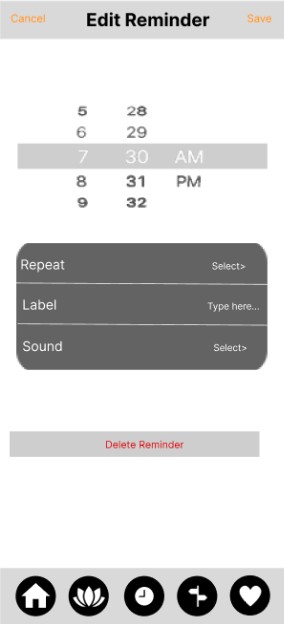


Share Data Page


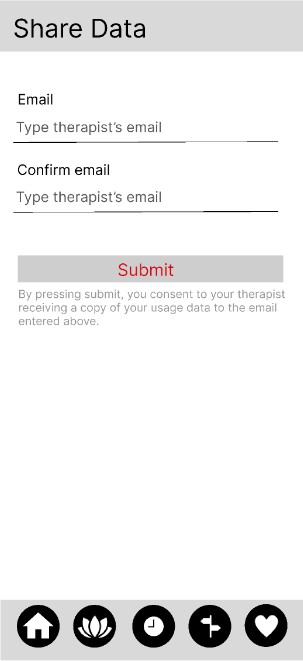


**Client Focus Group 3**

# ACTaide Wireframes


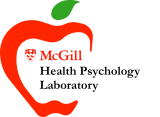


Library: All Skills Pages

Home Page

Library: Sort By Page


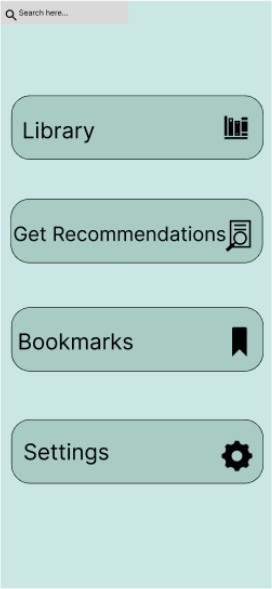

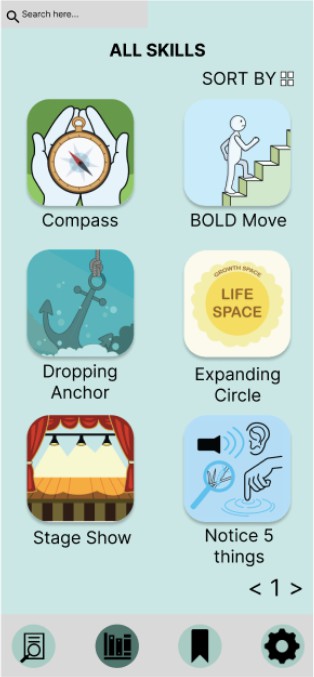

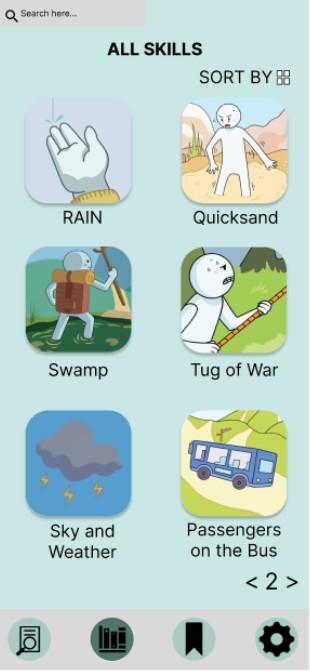

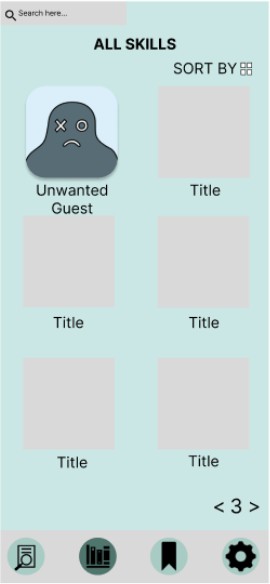

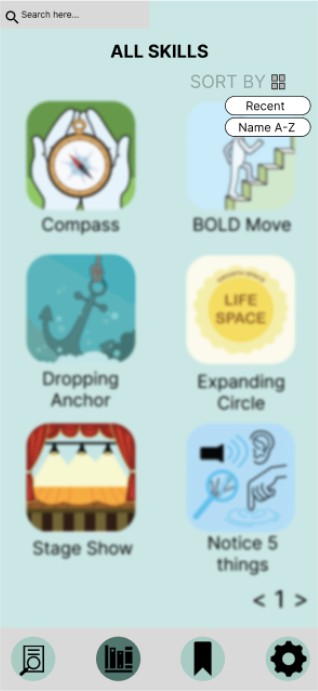


Assessment Pages: Hexaflex Questionnaire


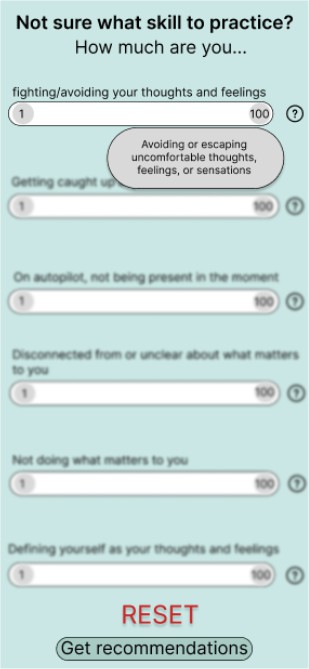

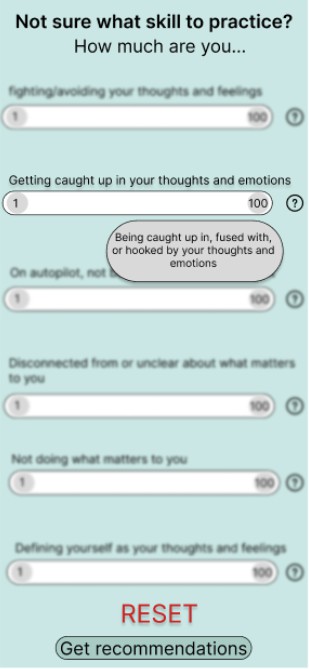

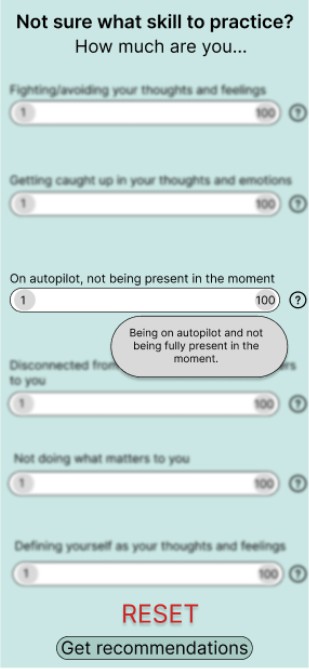


Assessment Pages: Hexaflex Questionnaire


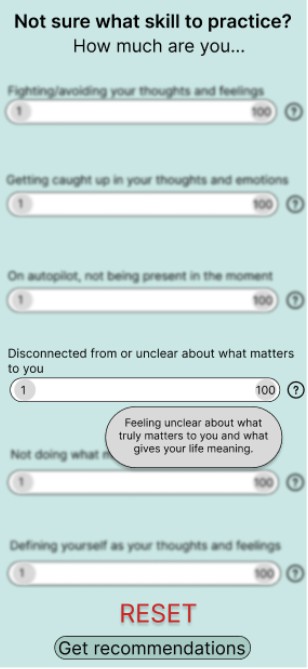

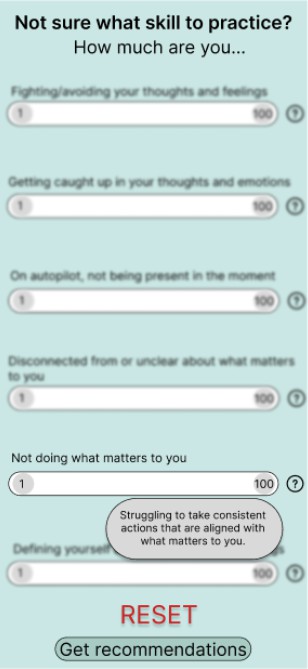

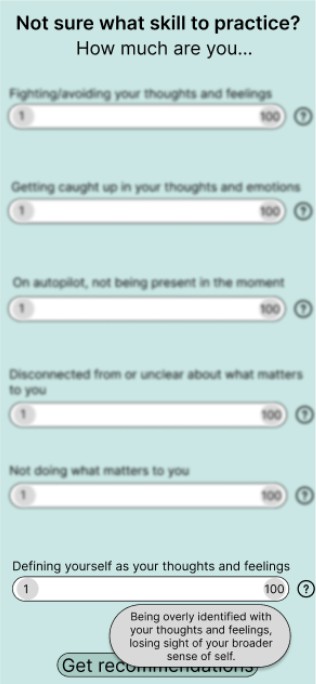


Assessment Pages: Triflex Questionnaire


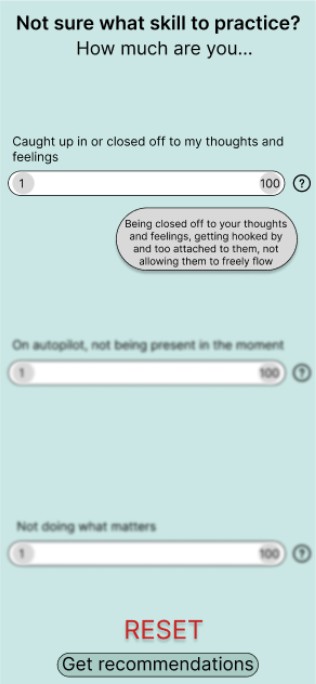

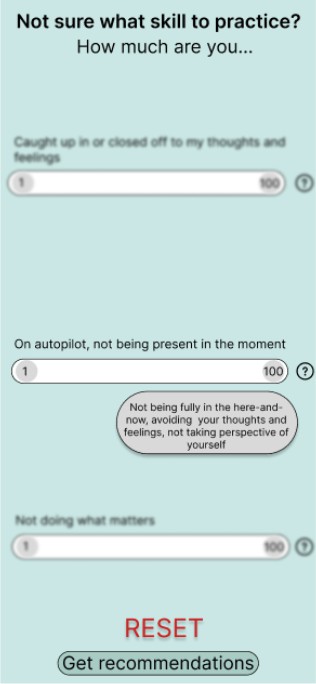

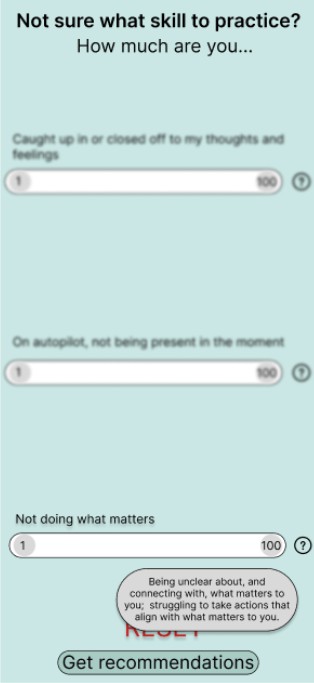


Bookmarks Page

Remove Bookmarks Page

Get Recommendations Page


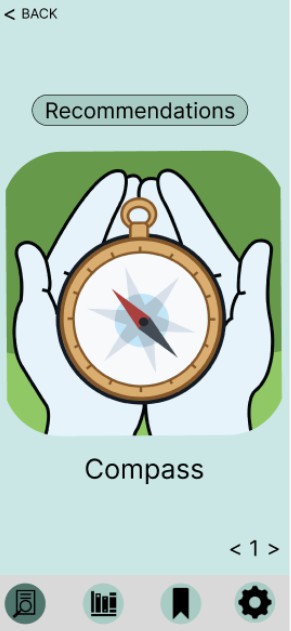

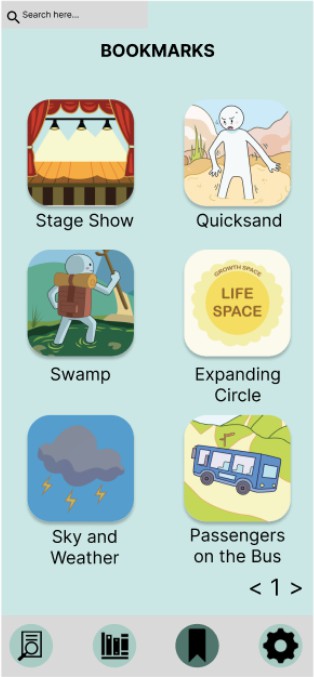

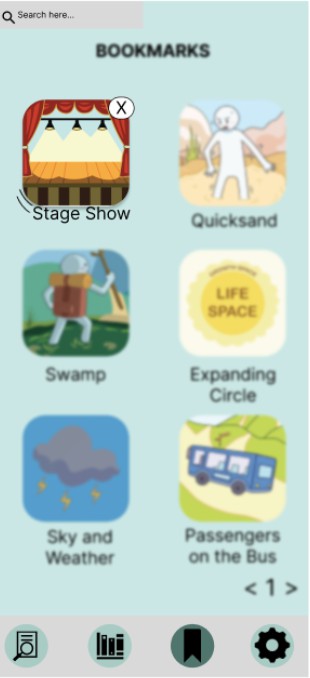


See More Page

Start Exercise Pages


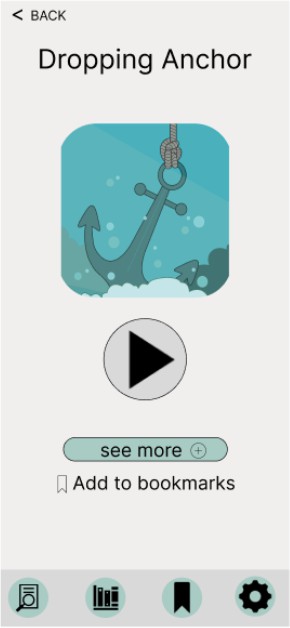

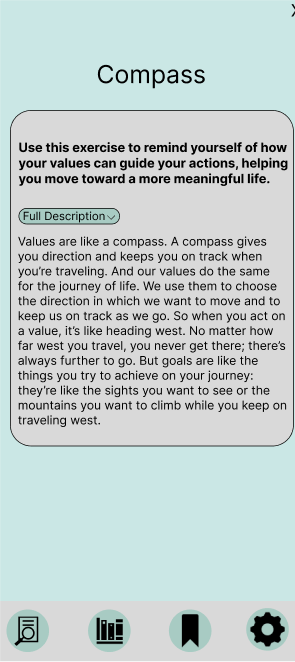


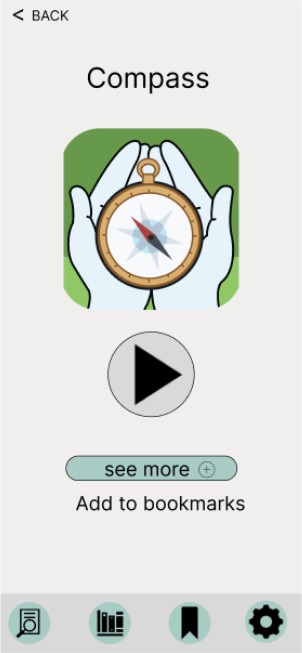

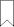

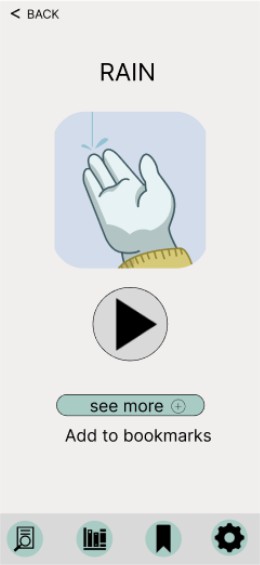

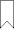


Annotated Image Sequence Pages: Dropping Anchor


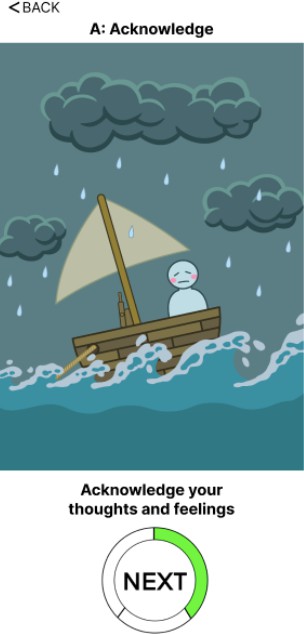

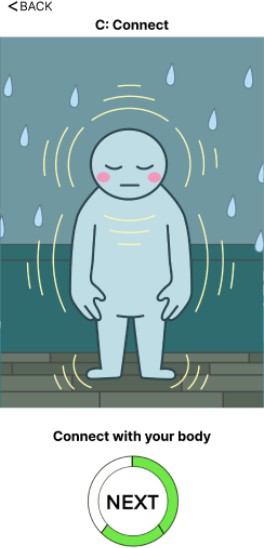

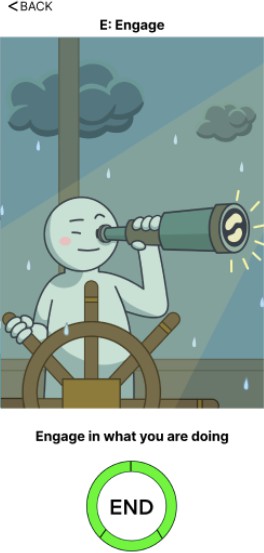


Annotated Image Sequence Pages: RAIN


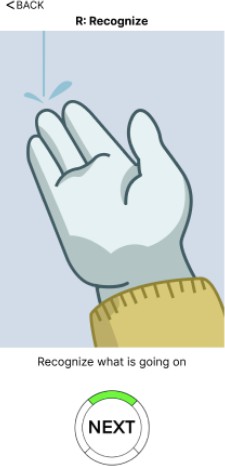

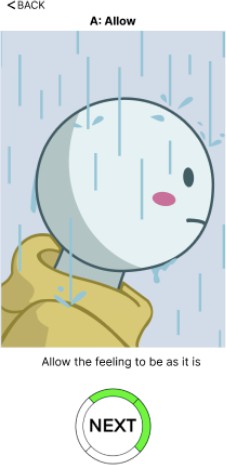

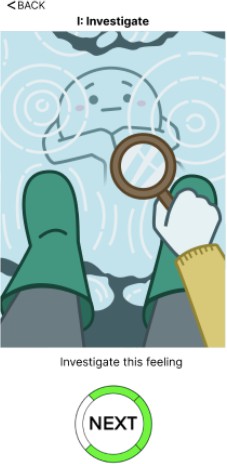

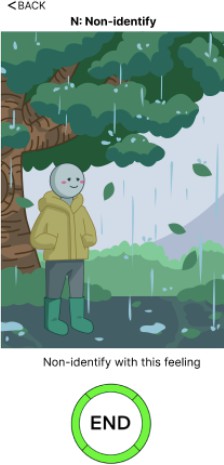


Annotated Image Sequence Pages: Compass


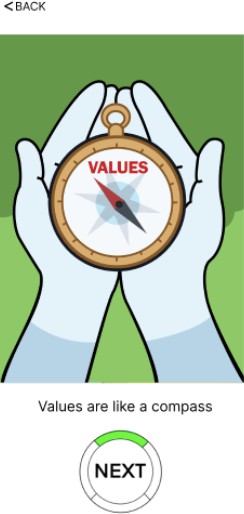

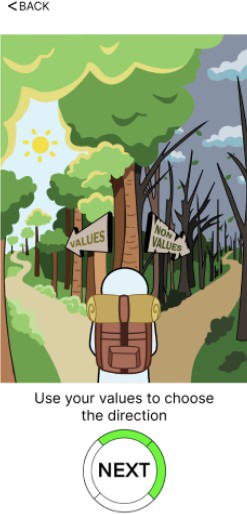

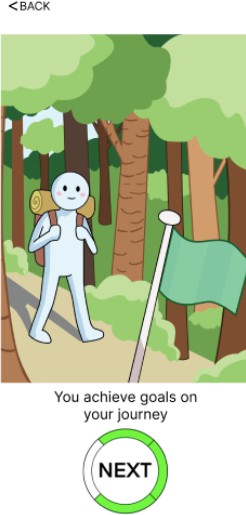

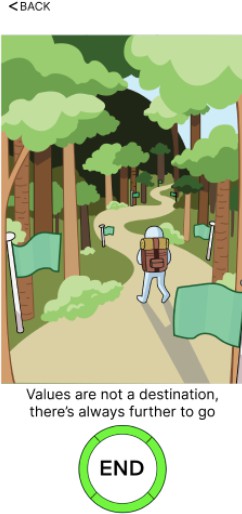


Add Reminder Page

Reminders Page

Edit Reminder Page

Settings Page


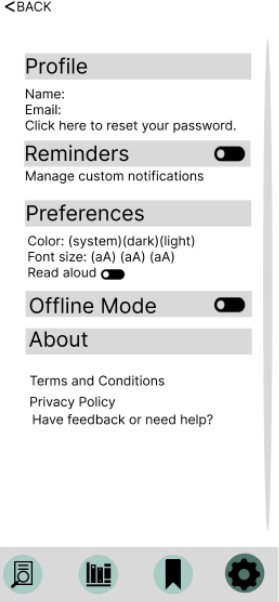

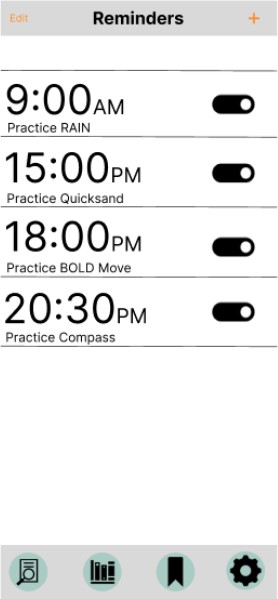

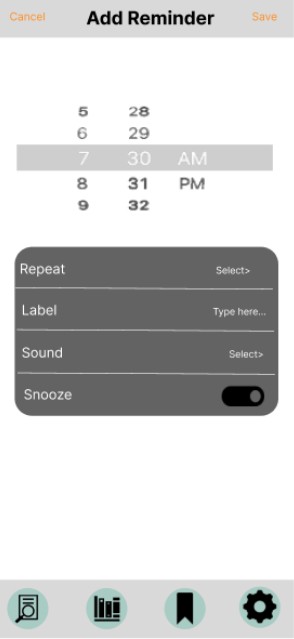

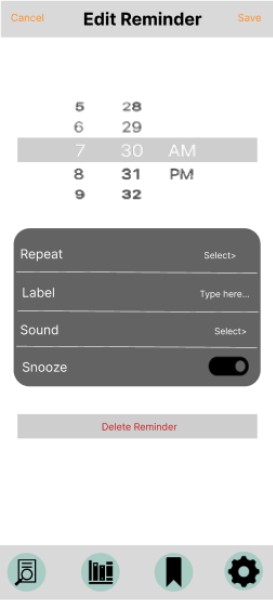


Snooze Option Pages


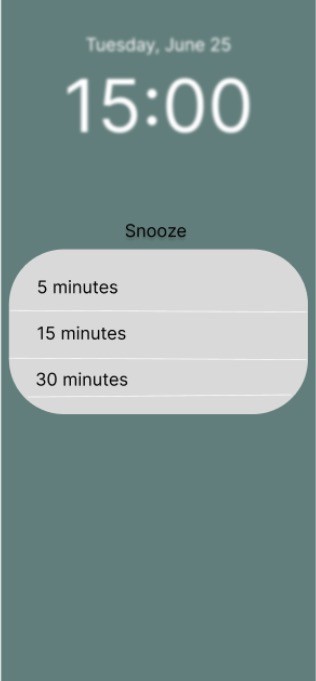

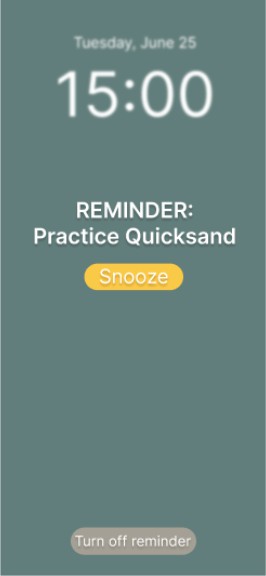

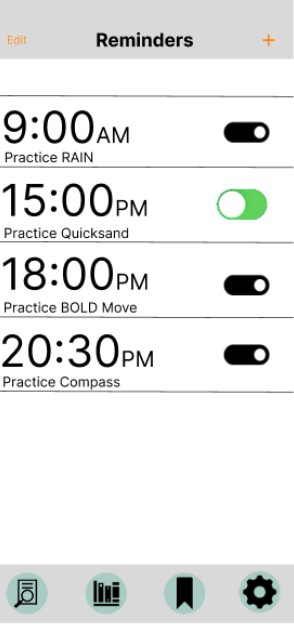

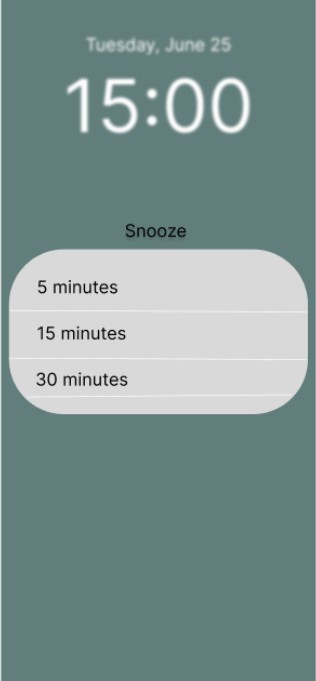


**Client Focus Group 4**

# ACTaide Wireframes


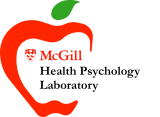


All Skills Pages

All Skills: Sort By Page

Home Page


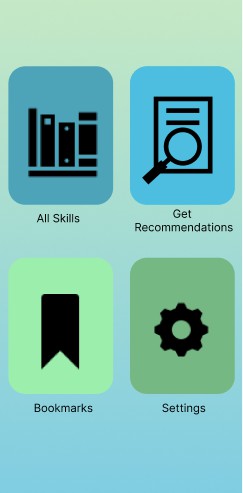

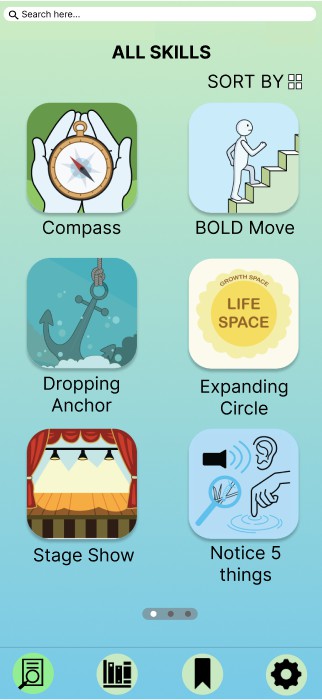

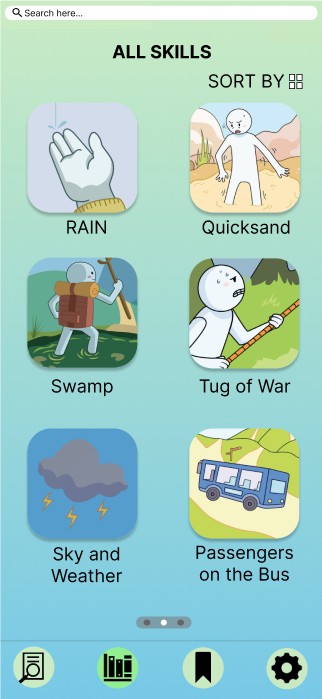

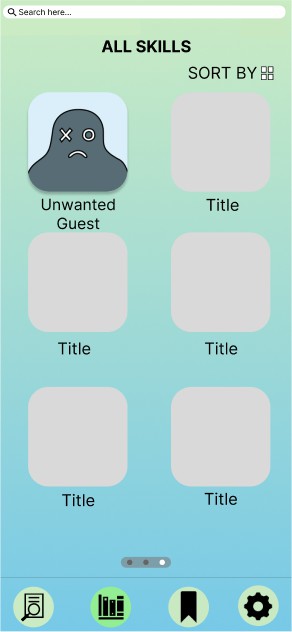

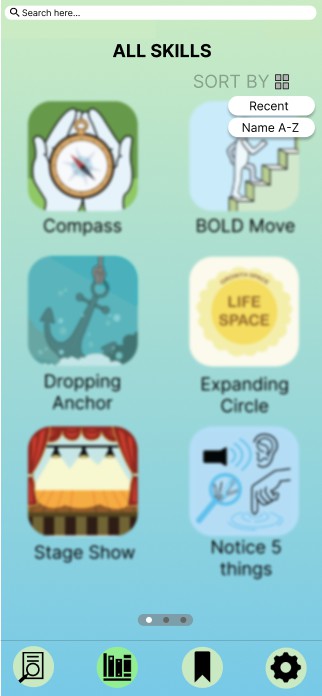


Assessment Pages: Hexaflex Questionnaire


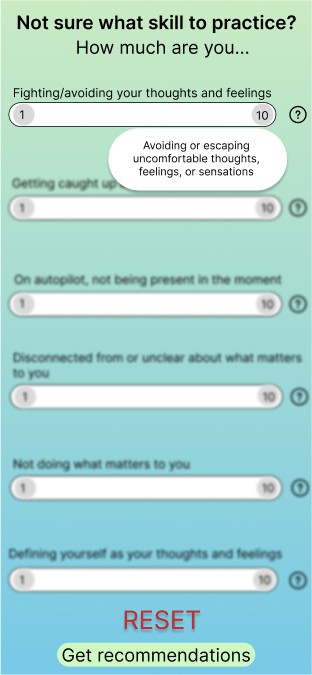

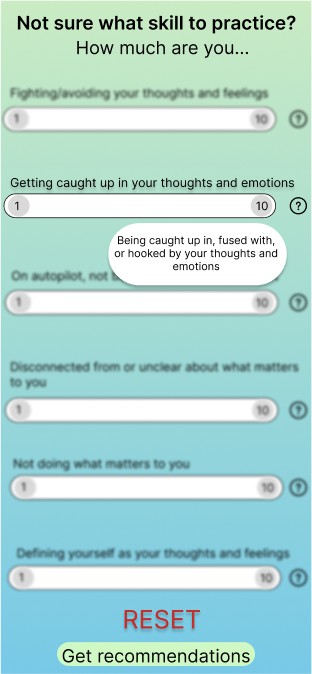

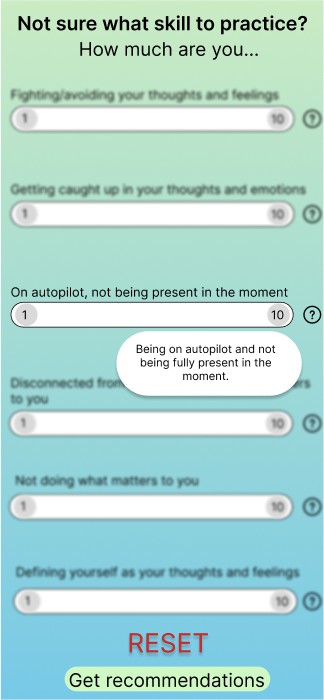


Assessment Pages: Hexaflex Questionnaire


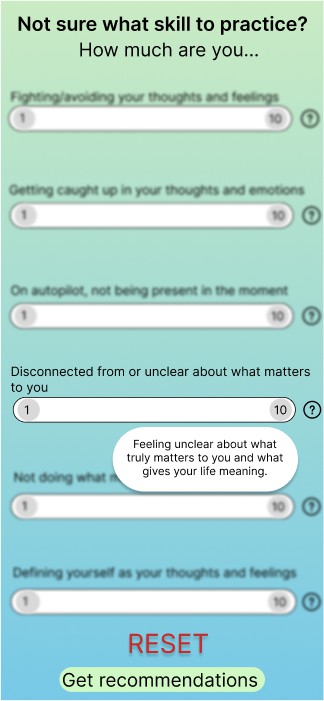


Assessment Pages: Triflex Questionnaire

Remove Bookmarks Page

Bookmarks Page

Get Recommendations Page

Start Exercise Pages

Learn More Page

Annotated Image Sequence Pages: Dropping Anchor

Annotated Image Sequence Pages: RAIN

Annotated Image Sequence Pages: Compass

Edit Reminder Page

Settings Page

Add Reminder Page

Reminders Page

Snooze Option
