# Supplementary material for: Therapy Companion Mobile App for Acceptance and Commitment Therapy Exercises (ACTaide): Therapist and Client Co-Design Study
Source: JMIR Form Res. 2025 Jul 24;9:e69532. doi: 10.2196/69532 (PMC12332461; doi:10.2196/69532)
Supplement: Multimedia Appendix 2 [file formative_v9i1e69532_app2.pdf]

Summary of Design Changes from Focus Group Feedback

Table S1. Changes made based on therapist feedback from the first focus group.

Wireframes	Feedback on App Component	Actions Taken
Home Page	• The drop-down menu is too academic (i.e., processes in the hexaflex are not always made explicit to clients)	• Remove "sort by" feature categorizing exercises by process
	• The home page is too busy and should be simplified	• Create three categories "open up, be present, do what matters" with drop-down menus for each<br>• Organize categories in the shape of a triangle
	• The names of some exercises are too long	• Simplify exercise names (e.g., compass, swamp)
Locked Exercise Page	• The unlock button does not have a margin around the text<br>• Feature with codes may impose therapist burden and is not necessary<br>• Feature introduces external motivation and gamification	• Remove the lock and codes features
Exercise Page Overview	• The back arrow is too minimal	• Replace icon with the word "back"
	• Estimated duration feature is not relevant as exercises can be done for variable amounts of time	• Remove estimated duration and retain progress bar

	• "About" and "Tags" are not necessary features	• Remove "about" and "tags" features
Distress Rating Scale Pages	• Unnecessary and not clinically relevant<br>• Perceived as conflicting with ACT philosophy due to its focus on symptomatology<br>• Concern that it might cause distress if no changes are observed	• Remove page
Breathe Page	• Breathing deeply is not always accessible to people<br>• Need a more universal anchor	• Change the wording from "breathe deeply" to "take a moment"
Exercise Page	• The start button does not have a margin around the text	• Add space around text
	• Dislike the term "start"	• Change "start" to "are you ready?"
	• The "see more" button available as one engages with an exercise can hinder full engagement	• Move the "see more" button to the exercise overview page
Settings Page	• The font is too small	• Add an option for increasing font size
	• Could be useful to obtain client usage data (i.e., time spent on app and on different exercises) but not realistic in clinical practice<br>• Sharing data feature with therapists should be optional	• Make sharing data feature optional
	• Desire for optional reminders (default is none)	• Create optional reminders for how many times a day they would like to be reminded and when (frequency and timing)

General	• Desire for a colored background<br>• Add more colors to the elements	• Add a background color (blue/green)<br>• Incorporate more colors

Table S2. Changes made based on therapist feedback from the second focus group.

Wireframes	Feedback on App Component	Actions Taken
Home Page	• The triangle shapes are not aesthetically appealing	• Remove triangles from the home page
	• The categories are too abstract and could be confusing for clients	• Remove Triflex categorization of exercises<br>• Retain categorization on the backend for algorithm
	• Dislike the term “favorites” given potential for promoting experiential avoidance	• Replace the word “favorites” with the word “bookmark”
List of Exercise Pages	• The list of exercises is too wordy and overwhelming	• Implementation of a visual-based interface where all exercises have a cover image
Start Exercise Page	• Start button as a circle with text is not visually appealing	• Replace button with the play icon
Take a Moment Page	• Asking clients to take a moment before doing the exercise is too abstract and may be an unnecessary step (too many clicks) prior to starting an exercise	• Remove feature
Reminders Pages	• Appreciation for this feature<br>• Interest in having a snooze option to delay reminders	• Add a snooze option

Share Data Page	• Dislike for this feature<br>• Adds workload to the therapists	• Remove feature
Exercise Page	• The progress bar should be in the shape of a circle to reflect the cyclical nature of many exercises	• Replacing progress bar with a looped progress cycle
	• There is too much blank space on the page	• Increase the size of the images

Table S3. Changes made based on client feedback from the third focus group.

Wireframes	Feedback on App Component	Actions Taken
Home Page	• The home page is plain and lacks contrast<br>• Not visually appealing<br>• Label “library” is not intuitive	• Replace rectangles with four quadrants in different colors to enhance contras<br>• Change background color<br>• Change “Library” label to “All Skills”
All Skills Pages	• Appreciation for the use of cover images<br>• Preference for widgets to show number of pages remaining<br>• Suggestion to reposition the search bar to span the entire width of the page	• Replace numerical digits with widget to indicate number of pages remaining<br>• Extend the search bar
Assessment Pages: Hexaflex Questionnaire	• Appreciation for the assessment pages<br>• Suggestion for a smaller scale (1-10)	• Change the scale to 1-10
Get Recommendations Page	• Suggestion to put both recommendations on a single page for simplicity	• Show both recommendations on one page
See More Page	• Label “see more” is not intuitive	• Change the label to “learn more”
Settings Page	• Suggestion to have a journal feature to reflect on a given exercise	• Add a journal feature
Snooze Page	• Interest in adding more snooze options	• Add a 24-hour snooze option
General	• The app’s color palette lacks sufficient contrast	• Incorporate a green and blue gradient to

	• Suggest using zen or calming colors	improve the contrast of the color palette

Table S4. Changes made based on client feedback from the fourth focus group.

Wireframes	Feedback on App Component	Actions to Be Taken
Home Page	• Unnecessary and complicates user experience	• Remove wireframe
All Skills Pages	• Label “all skills” is not intuitive<br>• Desire for progress tracking feature<br>• Desire to include personalization options, such as a greeting and using the user's name	• Label “all skills” changed to “exercises”<br>• Add goal-setting and progress tracking features<br>• Add greeting with user’s name
Assessment Pages: Hexaflex Questionnaire	• Concern about the similarity in wording between the items and their explanations<br>• Suggestion to redesign the sliding scale (rated 1 to 10) to be more visually appealing	• Rephrase the explanations of the items to avoid redundancy<br>• Redesign the sliding scale (e.g., adding color, haptic feedback, hexagon shaped)
Bookmarks Page	• Label “bookmarks” is not intuitive	• Replace label and icon “bookmark” with “favorites”
Start Exercise Page	• The “play” icon not intuitive as it suggests that a video will start	• Change the icon to an arrow with the word “start” in it
Learn More Page	• Dislike the pop-up<br>• Perceived as involving unnecessary clicking	• Provide one-sentence description of the exercise purpose on the start exercise page, with a dropdown to reveal the full-text
Exercise Pages	• Lack of interactivity (e.g., prompts and forced-choice questions) and not enough opportunities for engagement<br>• Desire for animations	• Increase interactivity by adding opportunities for users to input related to the exercises<br>• Add animations to images

Settings Page	• Reminders feature and journal feature are hidden and should be made more visible	• Add a journal icon to the navigation bar for easier access
Snooze Page	• Label “snooze” is not intuitive	• Replace label “snooze” with “delay”
General	• Options for personalization requested, such as color palette	• Include a selection of color schemes that users can choose from within the app settings
