# Supplementary material for: Therapy Companion Mobile App for Acceptance and Commitment Therapy Exercises (ACTaide): Therapist and Client Co-Design Study
Source: JMIR Form Res. 2025 Jul 24;9:e69532. doi: 10.2196/69532 (PMC12332461; doi:10.2196/69532)
Supplement: Multimedia Appendix 3 [file formative_v9i1e69532_app3.docx]

**Semi-Structured Focus Group Interview Guide**

**Therapist Focus Group**

Opening Question(s)

1. Could you please state your name, title, what setting you primarily work in, your primary patient population, and how long you have been using ACT in your clinical practice?

Introductory Question(s)

1. How do you incorporate the ACT exercises and metaphors into your clinical work?
2. What is your view on the role of homework or between-session practice in psychotherapy?

Transition Question(s)

1. What are your thoughts about the overall concept of the app presented?

Key Question(s)

1. What are your thoughts about and feedback on the current wireframe that was just presented?
2. What do you like about the app's design and functionality? What do you dislike about the app’s design and functionality?
3. Do you foresee any challenges that clients may experience navigating and using the app? How do you propose reducing barriers to use?
4. What features would you like to see added to the app? Are there any specific features that you think would be useful for your clients?
5. What features would you like to see removed from the app?
6. What modifications would need to be made to best support your clients in their therapy journey?
7. What are your thoughts about using the application to monitor your client’s progress and at home practice?
8. What kind of support would you as therapists need to use this app effectively?
9. What concerns, if any, do you have about the app?

Closing Question(s)

1. Of all the things we discussed, what is the most important to you and do you think we should take away from today's session?
2. Is there anything else that is important for you to share before we wrap up for today?

**Client Focus Group**

Opening Question(s)

1. Could you please tell us your name and what type of therapy you have done?

Introductory Question(s)

1. What has been the role of at-home exercises or homework in the therapy you have participated in?

Transition Question(s)

1. What are your thoughts about the overall concept of the app presented?

Key Question(s)

1. What are your thoughts about and feedback on the current wireframe that was just presented?
2. What do you like about the app's design and functionality? What do you dislike about the app’s design and functionality?
3. Do you foresee any challenges that you may experience navigating and using the app? How do you propose reducing barriers to use?
4. What features would you like to see added to the app? Are there any specific features that you think would be useful?
5. What features would you like to see removed from the app?
6. What modifications would need to be made to best support you in your therapy journey?
7. What concerns, if any, do you have about the app?

Closing Question(s)

1. Of all the things we discussed, what is the most important to you and do you think we should take away from today's session?
2. Is there anything else that is important for you to share before we wrap up for today?
